# Liquid‐Responsive Shape‐Memory Nanofiber‐Reinforced Scaffolds for Cartilage Repair

**DOI:** 10.1002/advs.77845

**Published:** 2026-09-27

**Authors:** Jiyang Zeng, Wei Li, Yawei Li, Zhiming Tu, Hong Ma, Yuliang Dai, Zhaoling Ma, Tao Yuan, Bing Wang

**Affiliations:** ^1^ Department of Spine Surgery Second Xiangya Hospital of Central South University Changsha China; ^2^ Hunan Digital Spine Research Institute Department of Science and Technology of Hunan Province Changsha China; ^3^ Hubei Key Laboratory of Biomass Resource Chemistry and Environmental Biotechnology Wuhan University Wuhan China; ^4^ Hubei International Scientific and Technological Cooperation Base of Sustainable Resource and Energy Wuhan University Wuhan China; ^5^ School of Resource and Environmental Science Wuhan University Wuhan China; ^6^ Guangxi Key Laboratory of Low Carbon Energy Materials Guangxi Normal University Guilin China

**Keywords:** BMSC migratory activity, cartilage regeneration, chondrogenic differentiation, core–shell nanofibers, liquid‐responsive shape memory, sustained kartogenin release

## Abstract

Bioengineered scaffolds hold promise for articular cartilage repair but are often limited by poor defect conformity, insufficient availability of endogenous reparative cells, and inadequate chondrogenic stimulation. Here, we developed a liquid‐responsive shape‐memory, core–shell nanofiber‐reinforced, directionally porous scaffold (QCG‐2%F/KGN) for endogenous cartilage regeneration. KGN‐loaded SF/PCL–PVA core–shell nanofibers were fabricated by coaxial electrospinning, fragmented, incorporated into a quaternized chitosan/gelatin matrix, and assembled by directional freeze‐casting. The aligned microchannels provided a structurally permissive route for cell infiltration and distribution, while the nanofiber network enhanced pore‐wall roughness, structural stability, water retention, and hydration‐triggered shape recovery. The core–shell fibers also enabled sustained KGN release over 30‐day period. In vitro, QCG‐2%F/KGN maintained high cell viability and promoted BMSC migratory activity, spreading, chondrogenic differentiation, and cartilage‐matrix deposition. In a rat full‐thickness cartilage‐defect model, the scaffold conformally filled the defect and enhanced hyaline cartilage like regeneration, accompanied by increased SOX9, COL2A1, and ACAN and decreased COL1A1 and MMP13. Transcriptomic analysis further indicated enrichment of cartilage‐anabolic programs and attenuation of inflammatory signaling. These findings support a sequential regenerative strategy integrating shape‐adaptive implantation, a microarchitecture favorable for endogenous cell infiltration, and sustained chondrogenic induction.

## Graphical abstract

A liquid‐responsive shape‐memory, nanofiber‐reinforced QCG scaffold combines conformal defect filling, an aligned architecture that is structurally permissive for endogenous cell infiltration, and sustained KGN release. This integrated design enhances BMSC viability, migratory activity, and chondrogenic differentiation and promotes hyaline cartilage‐like repair in a rat full‐thickness defect model.

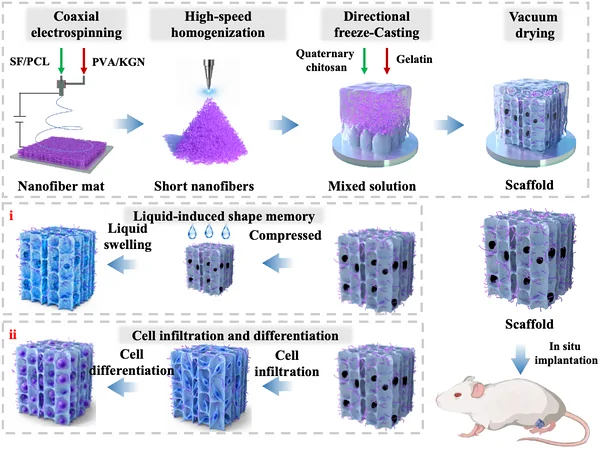

## 1 Introduction

Articular cartilage injury is a common cause of persistent joint pain, impaired mobility, and progressive disability, affecting more than 600 million people worldwide and imposing a substantial socioeconomic burden [1, 2]. Unlike most tissues, articular cartilage is avascular and aneural and contains sparsely distributed chondrocytes with limited proliferative activity [3, 4, 5]. Consequently, once damaged, it has little capacity to restore its highly organized extracellular matrix (ECM) or recover normal mechanical function [6, 7]. Current treatments may provide temporary improvement, but they recruit and retain few reparative cells and often generate mechanically inferior fibrocartilage [8, 9, 10, 11]. Bone marrow mesenchymal stem cells (BMSCs) residing in the subchondral region represent an attractive endogenous cell source because they can proliferate and differentiate toward the chondrogenic lineage [12]. Therefore, a regenerative platform that actively mobilizes endogenous BMSCs, retains them within the defect, and subsequently directs their chondrogenic differentiation would avoid the complexity of exogenous cell transplantation and may provide a more practical route toward functional cartilage regeneration.

Bioengineered scaffolds can serve not only as temporary defect fillers but also as instructive microenvironments that provide physical pathways and biochemical signals for endogenous repair. In particular, directionally porous scaffolds with aligned microchannels can connect the subchondral bone marrow cavity with the cartilage defect and thereby provide a continuous, relatively low‐resistance structural route that may facilitate endogenous cell infiltration and distribution [13, 14, 15, 16]. However, aligned porous scaffolds often have smooth, mechanically unstable pore walls and poor defect conformity. Their thin and smooth pore walls provide few anchoring interfaces for migrating cells and are susceptible to bending, wrinkling, or collapse under repeated compression, which compromises channel continuity and cell infiltration [17, 18]. Moreover, a preformed scaffold that does not adapt to the geometry of a defect may leave interfacial gaps or become unstable in the continuously loaded joint environment. Liquid‐responsive shape‐memory scaffolds offer a potential solution because they can be compressed into a smaller temporary configuration, placed into the defect, and subsequently recover their original dimensions after absorbing surrounding fluid, thereby achieving conformal filling and local fixation. However, introducing high porosity and hydration responsiveness often weakens mechanical stability. Three‐dimensional nanofiber networks are particularly suitable for overcoming this flaw: their interwoven topology can bridge adjacent pore walls, disperse concentrated stress [19, 20, 21, 22], and resist structural collapse, while their ECM‐like micro/nanotopography increases surface roughness and provides abundant adhesion sites for cell attachment, spreading, and migration [23, 24, 25]. Thus, integrating a reinforcing nanofiber network into a directionally porous, shape‐adaptive matrix may simultaneously improve defect conformity, mechanical resilience, and endogenous cell colonization. Enhanced cell infiltration alone does not ensure hyaline cartilage formation. Kartogenin (KGN), a stable small heterocyclic molecule, has attracted considerable attention because it can enhance BMSC migratory activity and promote chondrogenic differentiation, accompanied by increased expression of SRY‐box transcription factor 9 (SOX9), collagen type II alpha 1 chain (COL2A1), and aggrecan (ACAN) [26, 27, 28, 29]. Compared with protein growth factors, KGN also offers advantages including improved physicochemical stability, a longer functional lifetime, and a lower risk of immunogenicity [30, 31, 32]. Nevertheless, direct administration of free KGN may result in rapid diffusion from the defect and insufficient local exposure. Core–shell nanofibers can spatially isolate KGN within an inner phase, protect it during scaffold preparation, and provide sustained release as the fibers gradually hydrate and degrade [33, 34]. More importantly, when such fibers are used as the reinforcing skeleton of an aligned porous scaffold, the same structural component can provide mechanical reinforcement, cell‐adhesive topography, and prolonged chondrogenic stimulation. A scaffold combining shape recovery, a structure permissive for endogenous cell infiltration, and sustained chondrogenic induction is therefore desirable.

**FIGURE 1:**
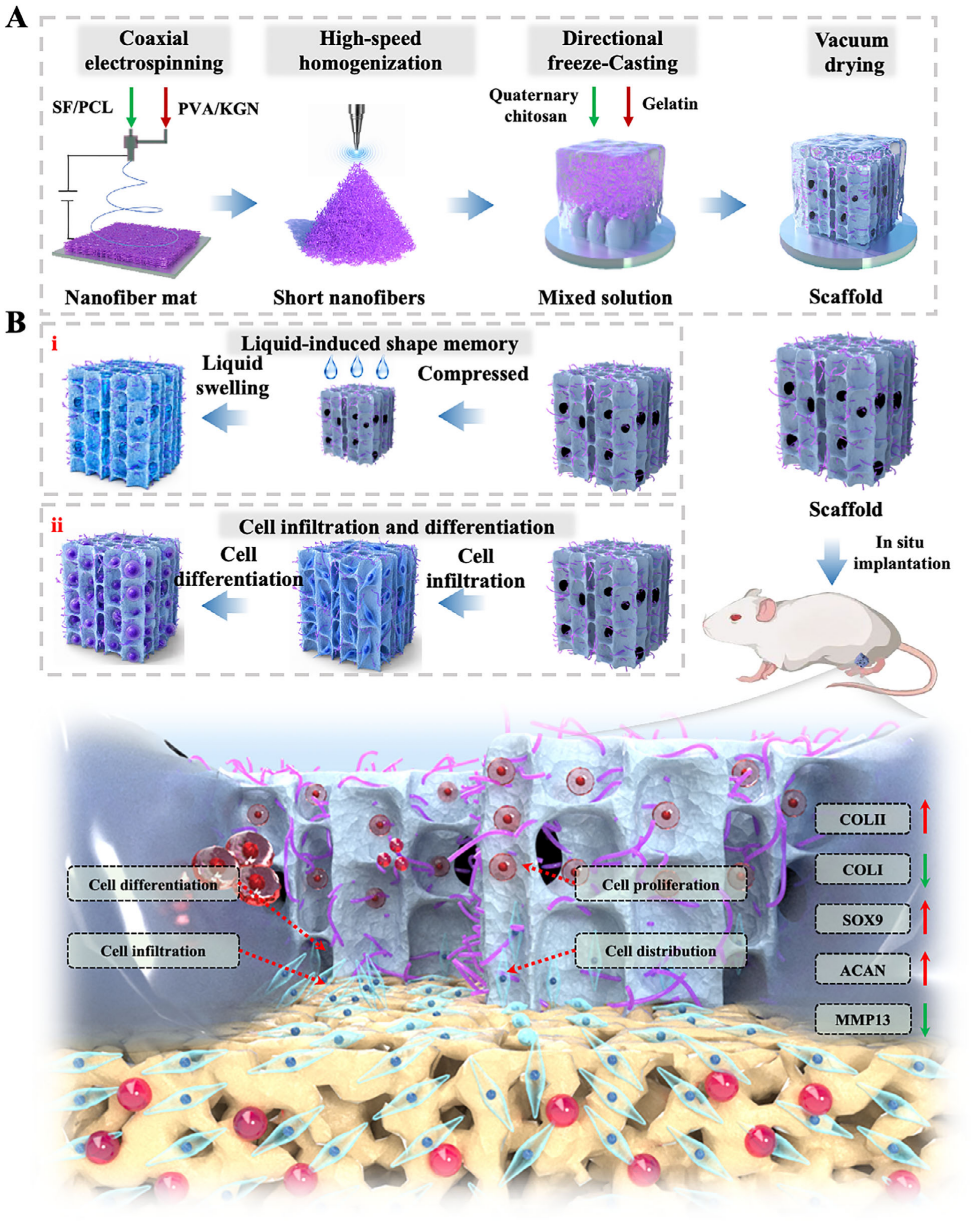
Schematic illustrating the fabrication strategy, multifunctional characteristics, and proposed endogenous‐cell‐assisted cartilage repair mechanism of the QCG‐2%F/KGN scaffold. (A) Core–shell nanofibers were first fabricated by coaxial electrospinning using an SF/PCL shell phase and a PVA/KGN core phase. The obtained nanofiber mats were fragmented into short nanofibers by high‐speed homogenization, dispersed within a quaternized chitosan/gelatin (QCG) matrix, and subsequently processed by directional freeze‐casting (DFC) and vacuum drying to generate a nanofiber‐reinforced scaffold with aligned porous microchannels. (B) The scaffold exhibits two complementary functional features. (i) Its liquid‐induced shape‐memory behavior enables compression into a smaller volume for minimally invasive placement, followed by rapid water uptake, shape recovery, swelling, and conformal filling of the cartilage defect. (ii) The aligned microchannels provide a continuous structural route that may favor endogenous cell infiltration and distribution, whereas the three‐dimensional nanofiber network increases cell‐adhesion interfaces and supports cell spreading and proliferation. Sustained local release of KGN further promotes chondrogenic differentiation, characterized by increased COL II, SOX9, and ACAN expression and reduced COL I and MMP13 expression. After implantation into a full‐thickness articular cartilage defect, these structural and biochemical cues act sequentially to enhance endogenous cell recruitment, chondrogenesis, extracellular‐matrix deposition, and hyaline cartilage‐like tissue regeneration.

Herein, we developed a liquid‐responsive, shape‐memory nanofiber‐reinforced scaffold with sustained KGN release (Figure 1). KGN‐loaded core–shell nanofibers composed of a silk fibroin/polycaprolactone (SF/PCL) shell and a poly(vinyl alcohol)/KGN (PVA/KGN) core were first prepared by coaxial electrospinning and subsequently fragmented into short fibers. Material selection was guided by the specific fabrication and functional requirements of this design. Gelatin provides a collagen‐derived, cell‐adhesive matrix but has limited structural stability in physiological aqueous conditions, whereas quaternized chitosan improves hydrophilicity and physicochemical integrity and is compatible with directional freezing. SF/PCL and PVA were selected for their fiber‐forming compatibility in coaxial electrospinning: the SF/PCL‐rich shell contributes structural reinforcement and a diffusion barrier, while the PVA/KGN core enables spatial confinement of KGN. Hyaluronic acid and chondroitin sulfate are highly relevant cartilage‐ECM components, but their pronounced hydration and comparatively limited intrinsic mechanical stability generally require additional crosslinking or reinforcement when used as primary structural matrices. Accordingly, the QCG‐SF/PCL/PVA combination was chosen as a practical balance among cytocompatibility, directional pore formation, mechanical stabilization, and sustained drug delivery rather than as an intrinsically superior or universally necessary material set. These fibers were dispersed within a quaternized chitosan/gelatin (QCG) matrix and assembled into a directionally porous scaffold through directional freeze‐casting (DFC), yielding the optimized QCG‐2%F/KGN scaffold. The aligned microchannels were designed as a continuous structural pathway potentially favorable for endogenous cell infiltration from the subchondral side, whereas the interpenetrating nanofiber network increased pore‐wall roughness, stabilized the channel architecture, and dispersed compressive stress. Upon contact with liquid, the compressed scaffold rapidly re‐expanded and conformed to the defect, while the core–shell fibers enabled prolonged local KGN release to promote chondrogenic differentiation of cells that entered or colonized the scaffold. In vitro experiments demonstrated favorable cytocompatibility, enhanced BMSC migratory activity, adhesion, spreading, and cartilage matrix production. In a rat full‐thickness cartilage‐defect model, the QCG‐2%F/KGN scaffold improved defect filling, hyaline cartilage‐like matrix deposition, and integration with surrounding tissue. Transcriptomic analysis further indicated coordinated enrichment of chondrogenic and matrix‐anabolic programs together with attenuation of inflammatory and catabolic signaling. Collectively, this multifunctional scaffold integrates shape‐adaptive implantation, structural guidance, and sustained biochemical induction into a sequential regenerative strategy, providing a promising biomaterial platform for endogenous cartilage repair.

## 2 Results and Discussion

### 2.1 Fabrication and Hierarchical Structure of the Nanofiber‐Reinforced Directionally Porous Scaffolds

**FIGURE 2:**
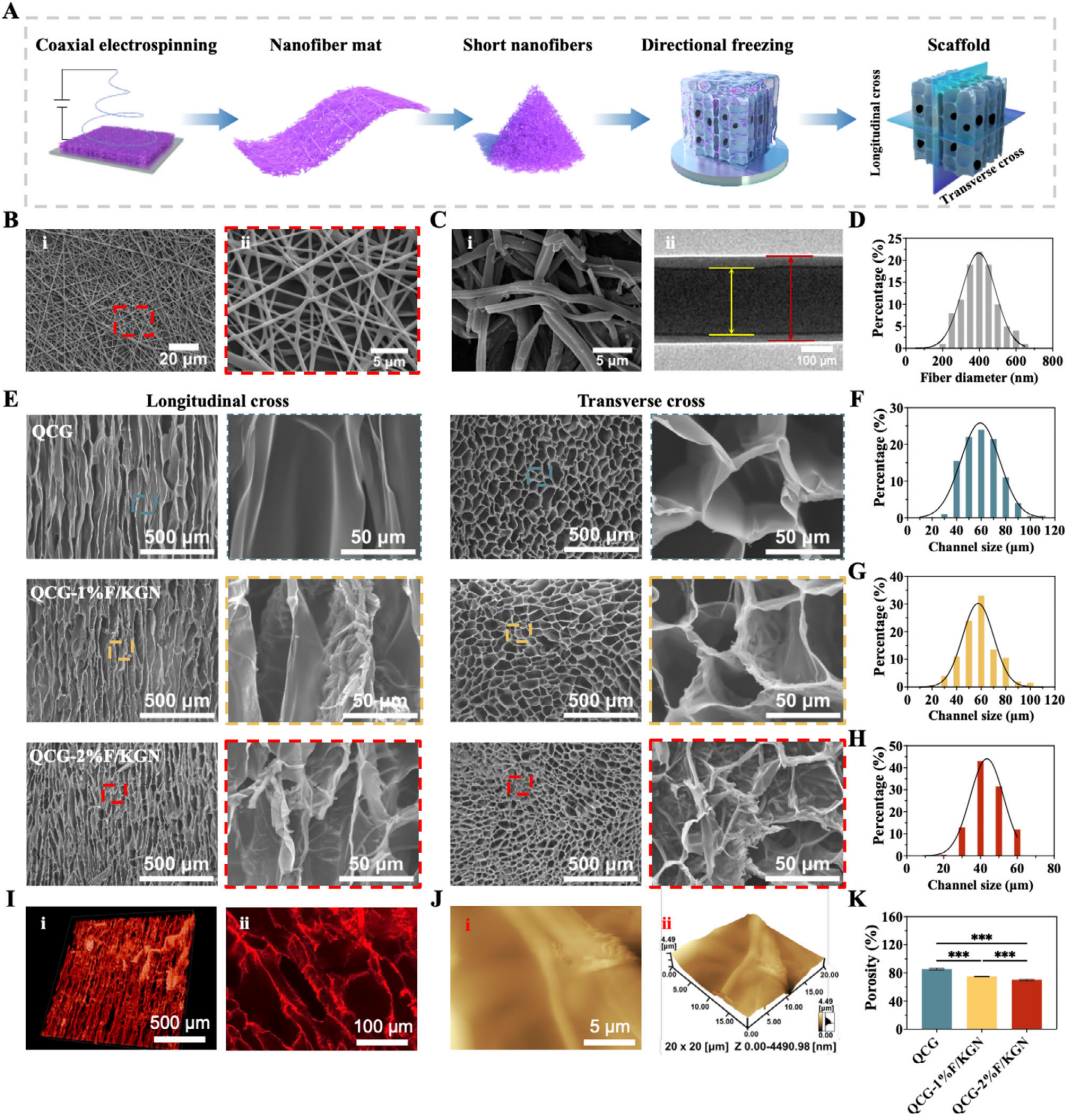
Hierarchical structural and morphological characterization of the nanofiber‐reinforced directionally porous scaffolds. (A) Schematic showing the preparation of the oriented scaffolds by coaxial electrospinning, collection of the nanofiber mat, high‐speed homogenization into short nanofibers, directional freezing, and vacuum drying; the longitudinal and transverse observation planes are indicated. (B) Representative low‐ and high‐magnification SEM images of the electrospun core–shell nanofiber mat, showing a uniform, bead‐free fibrous network. (C) Representative SEM image of the short nanofibers after homogenization (i) and TEM image showing the internal core–shell/hollow morphology of an individual nanofiber (ii). (D) Nanofiber diameter distribution, with diameters predominantly ranging from approximately 100 to 700 nm. (E) Representative low‐ and high‐magnification SEM images of longitudinal and transverse sections of QCG, QCG‐1%F/KGN, and QCG‐2%F/KGN scaffolds. Directional freezing generated continuous aligned channels in the longitudinal section and interconnected porous structures in the transverse section; nanofiber incorporation increased pore‐wall roughness and reduced wall wrinkling. (F‐H) Channel‐size distributions of the (F) QCG, (G) QCG‐1%F/KGN, and (H) QCG‐2%F/KGN scaffolds. (I) Three‐dimensional confocal fluorescence reconstruction and corresponding enlarged image showing the distribution of short nanofibers within the QCG‐2%F/KGN scaffold, including fibers embedded in the pore walls and fiber aggregates bridging adjacent channels. (J) Two‐dimensional and three‐dimensional AFM topographic images of the QCG‐2%F/KGN scaffold, demonstrating a roughened pore‐wall surface after nanofiber incorporation. (K) Porosity of the different scaffolds. Scale bars: 20 µm and 5 µm in (B); 5 µm and 100 µm in (C); 500 µm and 50 µm in (E); 500 µm and 100 µm in (I); and 5 µm in the two‐dimensional AFM image in (J). Quantitative data are presented as mean ± SD (n = 5 independent scaffold samples). One‐way ANOVA followed by Tukey's multiple‐comparison test was used for multi‐group quantitative comparisons; ^**^ *p* < 0.01, ^***^ *p* < 0.001; ns, not significant (*p* > 0.05).

The overall fabrication route is summarized in Figure 2A. KGN‐loaded core–shell nanofibers were first produced by coaxial electrospinning, collected as continuous fibrous mats, and then fragmented into short fibers by high‐speed homogenization. The short fibers were uniformly dispersed in the QCG precursor, after which directional freezing and vacuum drying converted the mixture into a three‐dimensional scaffold containing aligned porous channels. This multistep process was designed to integrate three functions within a single material: the directionally frozen QCG matrix provided a continuous structural pathway for cell infiltration, the short nanofibers acted as a reinforcing and cell‐adhesive skeleton, and the fiber core served as a reservoir for sustained KGN delivery.

Scanning electron microscopy (SEM) demonstrated that the electrospun mat consisted of a dense, randomly interwoven network of smooth fibers without obvious beads, fused regions, or gross diameter irregularities (Figure 2B). The absence of bead‐like defects suggests that the shell and core solutions were delivered under a stable electrospinning regime. Quantitative analysis of the diameter distribution (Figure 2D) showed that most fibers were between approximately 100 and 700 nm, with an average diameter of 379.13 ± 92.97 nm. This submicrometer scale is advantageous for constructing an ECM‐like interface because it substantially increases the available surface area without occupying the macroscopic channels required for cell infiltration.

After homogenization, SEM confirmed that the continuous mat was effectively converted into discrete short nanofibers while the fibrous morphology was retained (Figure 2C(i)). Transmission electron microscopy (TEM) further revealed a clear internal core–shell/hollow architecture in individual fibers (Figure 2C(ii)). Although TEM alone does not directly quantify KGN loading, the distinct compartmentalized structure verifies successful formation of a protected inner domain capable of accommodating the PVA/KGN phase. Retention of this architecture after homogenization is important because excessive fragmentation or shell rupture would be expected to cause premature KGN leakage and reduce the duration of local biochemical stimulation [35].

The effect of nanofiber incorporation on scaffold architecture was next evaluated in both longitudinal and transverse sections (Figure 2E). In the longitudinal direction, all three formulations exhibited continuous channels aligned with the imposed freezing direction, confirming that the addition of short fibers did not disrupt directional ice‐crystal growth. The nanofiber‐free QCG scaffold displayed comparatively smooth channel walls. In contrast, QCG‐1%F/KGN and QCG‐2%F/KGN showed progressively rougher walls, with numerous fiber‐like features exposed on or embedded within the matrix. The roughness was most evident in QCG‐2%F/KGN, consistent with its higher fiber content. These aligned channels are expected to reduce the tortuosity encountered by BMSCs migrating from the subchondral region, whereas the roughened surfaces provide additional anchoring points for repeated attachment and traction generation during migration.

Transverse SEM images provided complementary information on channel continuity and wall stability (Figure 2E). QCG exhibited an interconnected porous network, but many pore walls were curved, folded, or locally wrinkled. The walls became straighter and more clearly supported after fiber incorporation, particularly in QCG‐2%F/KGN. This difference likely arises because the dispersed fibers become entrapped within the polymer‐rich regions between advancing ice crystals and subsequently act as internal braces during freezing and drying. By restraining local shrinkage and bending, the fibers reduce the probability of wall collapse and preserve communication between adjacent channels. Such stabilization is especially relevant after implantation, because loss of channel continuity would impede both cell entry and nutrient transport.

Channel‐size analysis was consistent with the morphological observations. The QCG channels were mainly distributed between 20 and 120 µm and had an average diameter of 55.38 ± 14.47 µm (Figure 2F). QCG‐1%F/KGN showed a similar principal range and an average diameter of 53.39 ± 14.33 µm (Figure 2G), whereas QCG‐2%F/KGN exhibited a narrower distribution concentrated mainly below 60 µm, with an average diameter of 38.71 ± 8.28 µm (Figure 2H; Figure S1). The progressive reduction and narrowing of the size distribution may reflect the increased solid content and viscosity of the precursor together with physical interference of the fibers with lateral ice‐crystal growth. Importantly, the reduction in channel size did not eliminate the open architecture [36, 37]. Porosity remained above 70% in all groups, although it decreased significantly with increasing fiber content (Figure 2K). Thus, QCG‐2%F/KGN achieved a useful compromise between structural reinforcement and preservation of sufficient void space for cell infiltration, fluid exchange, and diffusion of soluble factors.

Three‐dimensional confocal fluorescence reconstruction directly visualized the spatial distribution of the short fibers in QCG‐2%F/KGN (Figure 2I). The fibers appeared in two complementary configurations: dispersed fibers were incorporated into individual pore walls, while small fiber bundles extended across or connected neighboring channels. The corresponding nanofiber‐free QCG scaffold retained the aligned porous framework but lacked these bridging structures (Figure S2). The dispersed fibers increase the local cell‐contact area, whereas the bridging bundles create a secondary load‐transfer pathway across the porous matrix. This dual distribution explains how a relatively low nanofiber fraction can influence both cell‐scale topography and bulk mechanical behavior.

Atomic force microscopy (AFM) further quantified the change in surface topography. The pore‐wall height variation was approximately 1.19 µm for nanofiber‐free QCG (Figure S3) but increased to approximately 4.49 µm after incorporation of 2% fibers (Figure 2J). The three‐dimensional AFM map also showed distinct protrusions and ridges rather than the relatively smooth surface observed in QCG. This increase in micro/nanoscale roughness is likely to enhance protein adsorption and focal cell‐substrate contacts, which is consistent with the improved BMSC spreading and migration observed in subsequent experiments. Taken together, Figure 2 demonstrates that the fabrication strategy produced a hierarchical scaffold in which aligned macroscopic channels, interconnected micropores, roughened fiber‐containing walls, and interchannel fiber bridges operate at different length scales to support endogenous cell infiltration and colonization while maintaining structural integrity.

### 2.2 Liquid‐Induced Shape Memory, Hydration Behavior, Sustained KGN Release, and Mechanical Reinforcement

**FIGURE 3:**
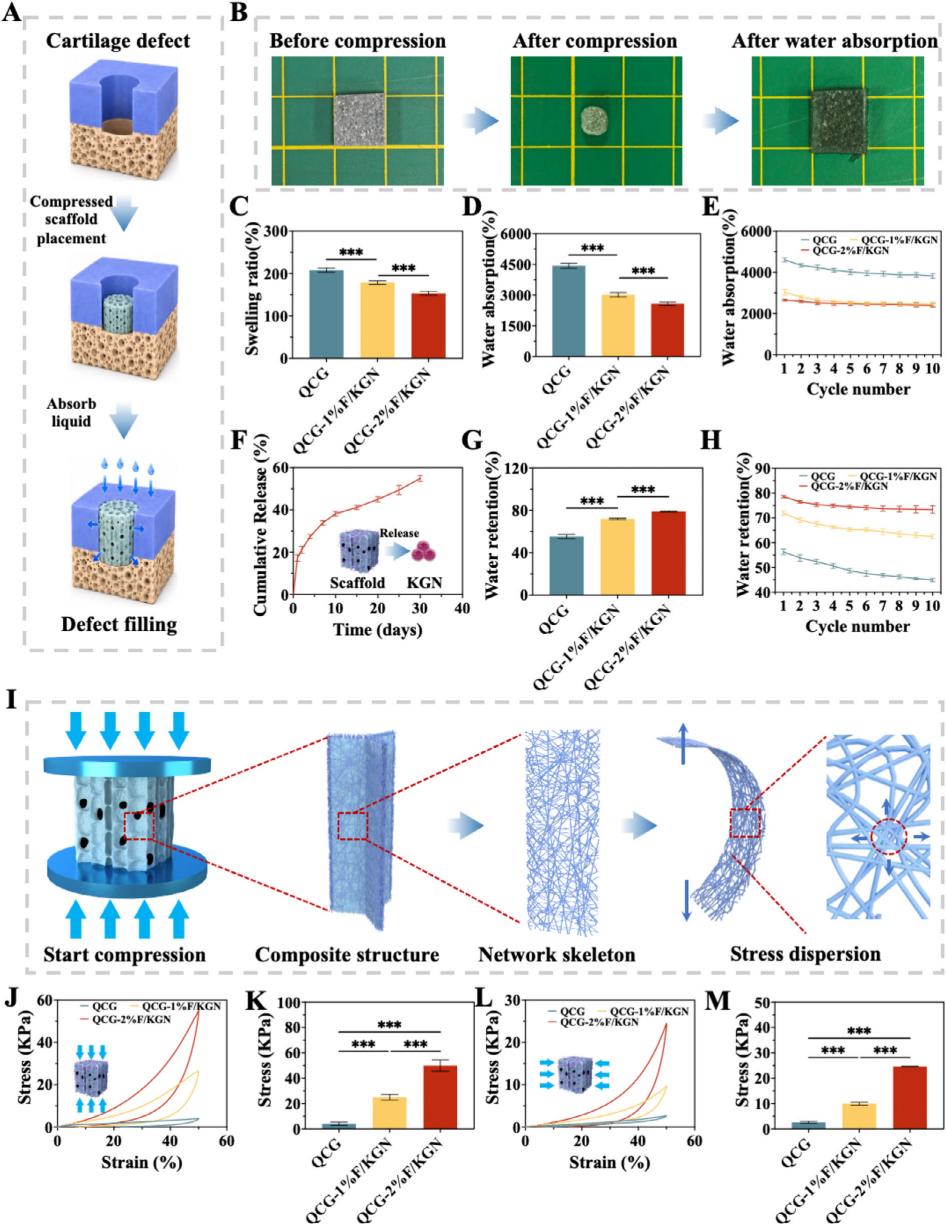
Liquid‐responsive shape recovery, water‐related properties, sustained KGN release, and wet‐state mechanical performance of the scaffolds. (A) Schematic showing the minimally invasive implantation concept: a compressed scaffold is inserted into a full‐thickness cartilage defect and subsequently absorbs surrounding fluid, recovers its original shape, and expands to fill and stabilize within the defect. (B) Photographs of a QCG‐2%F/KGN scaffold before compression, after compression, and after water absorption, demonstrating liquid‐induced shape recovery. (C) Volumetric swelling ratios of QCG, QCG‐1%F/KGN, and QCG‐2%F/KGN scaffolds after hydration. (D) Water‐absorption capacities of the three scaffolds. (E) Cyclic water‐absorption behavior during repeated compression and rehydration, showing improved stability after nanofiber incorporation. (F) Cumulative KGN‐release profile of the QCG‐2%F/KGN scaffold over 30 days, demonstrating an initial release phase followed by sustained release. (G) Water‐retention ratios after centrifugation. (H) Cyclic water‐retention behavior during repeated dehydration and rehydration. (I) Proposed nanofiber‐reinforcement mechanism during compression. Short nanofibers form an interpenetrating network within the porous matrix, restrain pore‐wall bending, bridge local structural units, and disperse concentrated stress at pore‐wall junctions. (J,K) Wet‐state compressive stress‐strain curves in the longitudinal direction and corresponding maximum compressive stress. (L,M) Wet‐state compressive stress‐strain curves in the transverse direction and corresponding maximum compressive stress. Quantitative data are presented as mean ± SD (n = 5 independent scaffold samples). One‐way ANOVA followed by Tukey's multiple‐comparison test was used for multi‐group quantitative comparisons; ^***^ *p* < 0.001; ns, not significant (*p* > 0.05).

The liquid‐responsive implantation concept is illustrated in Figure 3A. The dry scaffold could be compressed into a substantially smaller temporary configuration, placed within a confined full‐thickness cartilage defect, and then exposed to surrounding fluid. The photographs in Figure 3B show that QCG‐2%F/KGN retained the compressed state before hydration but rapidly expanded toward its original geometry after water absorption. This response demonstrates liquid‐induced shape‐memory behavior rather than simple passive swelling: compression enables placement, while hydration provides the driving force for dimensional recovery and conformal contact with the defect wall. The recovery most likely results from the combined elastic recoil of the crosslinked porous framework and osmotic hydration of hydrophilic QCG/gelatin chains.

Volumetric swelling measurements quantified the extent of hydration‐induced expansion (Figure 3C). The swelling ratios were 207.92 ± 4.95% for QCG, 178.81 ± 4.24% for QCG‐1%F/KGN, and 153.38 ± 4.58% for QCG‐2%F/KGN. Thus, every scaffold expanded markedly after hydration, but the magnitude decreased as the fiber fraction increased. The lower swelling of the reinforced formulations may be attributed to two related effects: the SF/PCL‐containing fibers impose physical constraints on expansion of the QCG network, and the relatively hydrophobic PCL component reduces the effective water affinity of the composite. Moderate rather than unrestricted swelling is desirable because excessive expansion could distort the defect margin or destabilize newly forming tissue, whereas the measured expansion remained sufficient to recover the original scaffold volume and fill the defect.

Water‐absorption capacity showed the same fiber‐dependent trend (Figure 3D). QCG absorbed 4434.71 ± 118.87% of its dry mass, whereas QCG‐1%F/KGN and QCG‐2%F/KGN absorbed 3015.09 ± 105.19% and 2579.21 ± 76.28%, respectively. Although incorporation of fibers significantly reduced total uptake, even QCG‐2%F/KGN absorbed more than 25 times its dry mass. This high capacity indicates that the scaffold retained an extensively interconnected aqueous phase capable of supporting diffusion of nutrients, metabolites, and soluble signals. The reduced uptake should therefore be interpreted as controlled hydration rather than loss of permeability.

Because an implanted scaffold experiences repeated compression and rehydration during joint motion, water absorption was also measured over multiple cycles (Figure 3E). QCG showed a pronounced progressive loss of absorption capacity, whereas both nanofiber‐containing scaffolds displayed smaller reductions and more stable curves. The difference suggests that repeated loading irreversibly damaged or compacted portions of the unsupported QCG pore network. In the reinforced groups, fibers embedded in and between the walls limited permanent buckling, enabling the channels to reopen during rehydration. QCG‐2%F/KGN therefore combined a lower initial uptake with greater functional stability, which may be more relevant in vivo than a high first‐cycle value that rapidly declines.

The KGN‐release profile was then evaluated over 30 days (Figure 3F). QCG‐2%F/KGN showed a relatively rapid early phase followed by a slower, sustained phase, and the cumulative release reached 54.9% (a blank‐corrected estimate under the specified experimental conditions) at day 30. The initial fraction can reasonably be assigned to KGN located near damaged fiber ends or transferred from partially fragmented fibers into the surrounding QCG matrix during homogenization and scaffold fabrication. Once this accessible fraction had diffused out, further release was governed by water penetration through the shell and gradual hydrolysis or fragmentation of the core–shell fibers. The incomplete release at 30 days indicates that a substantial fraction remained protected within the fibers, supporting prolonged local exposure rather than a short burst.

The degradation profile provides additional evidence for this interpretation (Figure S4). QCG‐2%F/KGN retained approximately 99% of its initial mass on day 1 and approximately 95% during the first 10 days, followed by gradual mass loss to approximately 80% at day 30. The scaffold therefore degraded slowly without abrupt disintegration during the period in which KGN was being released. Because release reached 54.9% while only about 20% of total scaffold mass was lost, drug liberation cannot be explained solely by bulk erosion. Instead, diffusion, local fiber hydrolysis, and progressive exposure of the PVA/KGN core jointly controlled the release process. This temporal pattern is well suited to a sequential repair strategy: endogenous cells can first enter and colonize the scaffold, after which sustained KGN signaling continues to promote chondrogenic differentiation and matrix synthesis.

Water retention after external force was distinct from total water uptake. Following centrifugation, the retention ratios were 55.31 ± 1.96% for QCG, 71.92 ± 0.69% for QCG‐1%F/KGN, and 78.87 ± 0.59% for QCG‐2%F/KGN (Figure 3G). Thus, the most highly reinforced scaffold absorbed less water overall but retained a larger proportion of the absorbed fluid. A plausible explanation is that the fibers stabilized small capillary spaces and limited irreversible collapse of the water‐holding network, whereas the unsupported QCG matrix released fluid more readily when deformed. Repeated dehydration and rehydration produced the same ranking (Figure 3H): QCG declined continuously, QCG‐1%F/KGN showed an intermediate response, and QCG‐2%F/KGN maintained the highest and most stable retention. Stable hydration should help preserve nutrient transport and prevent repeated drying of the cell‐material interface during cyclic joint loading.

The proposed nanofiber‐reinforcement mechanism is illustrated in Figure 3I. Under compression, the porous walls bend and transmit stress toward wall junctions. In QCG alone, these thin walls are susceptible to buckling and local stress concentration. In the composite scaffold, short fibers embedded in the walls act as tensile restraints, while fiber bundles spanning adjacent structural units distribute load over a larger area. The fibers therefore function as an interpenetrating secondary skeleton that opposes wall bending, bridges local defects, and protects junctions from collapse. This mechanism is consistent with the straighter pore walls observed by SEM and the superior cyclic hydration behavior.

Wet‐state compression testing confirmed a strong fiber‐dependent increase in load‐bearing capacity (Figure 3J–M). In the longitudinal test set, the stress‐strain curves shifted progressively upward from QCG to QCG‐1%F/KGN and QCG‐2%F/KGN (Figure 3J), and the corresponding maximum stress increased from 4.02 ± 1.29 kPa to 49.95 ± 4.46 kPa (Figure 3K). In the transverse test set, a similar graded increase was observed (Figure 3L), with the maximum stress rising from 2.60 ± 0.41 kPa to 24.60 ± 0.14 kPa (Figure 3M). The different stress levels in the two orientations demonstrate the expected anisotropy of a directionally frozen scaffold. More importantly, the substantial increase in both orientations shows that reinforcement was not limited to one loading axis; the dispersed fibers improved the integrity of the entire porous network.

Dry‐state testing showed the same overall dependence on fiber content (Figure S5). The maximum compressive stress increased from 307.13 ± 18.56 to 831.52 ± 58.63 kPa in the longitudinal orientation and from 108.87 ± 5.94 to 420.03 ± 8.11 kPa in the transverse orientation. The large difference between dry‐ and wet‐state values reflects plasticization of the QCG/gelatin matrix after hydration, which is necessary for rapid shape recovery and tissue conformability. An additional 3 wt% fiber formulation was also mechanically screened to examine whether further fiber addition would continuously improve performance. The 3% scaffold did not show a uniformly improved deformation response; a pronounced fluctuation/discontinuity appeared as compression approached approximately 50% strain, consistent with local structural damage or failure. By comparison, the 2% formulation showed a smoother response over the same compression range (Figure S6). To address early‐stage mechanical durability under repeated deformation, additional wet‐state cyclic‐compression screening was performed over 500 cycles. QCG‐2%F/KGN maintained a stable cyclic response without obvious macroscopic collapse or fragmentation, whereas the nanofiber‐free QCG scaffold showed greater progressive structural deterioration. These findings support the role of the interpenetrating fiber network in improving fatigue resistance and preserving the porous framework (Figure S7). It must be acknowledged, however, that the simplified compression test does not reproduce the combined compression, shear, friction, and multidirectional loading present in an articulating joint. The current data therefore support repeated‐deformation tolerance rather than mechanical equivalence to the load‐bearing capacity of native cartilage. Although the wet‐state strength of 24.60–49.95 kPa remains below the megapascal‐scale compressive properties of mature native cartilage, the present scaffold is intended as a temporary regenerative template rather than a permanent cartilage substitute. Its relevant functions are to survive implantation, remain open during early tissue ingrowth, recover after deformation, and progressively transfer load to newly formed matrix. The combination of compressibility in the dry state, liquid‐triggered recovery, high fluid retention, sustained KGN release, and reinforced wet‐state stability therefore provides a coherent physical basis for defect filling and early‐stage cartilage regeneration.

### 2.3 QCG‐2%F/KGN Maintains BMSC Viability and Enhances Migratory Activity In Vitro

**FIGURE 4:**
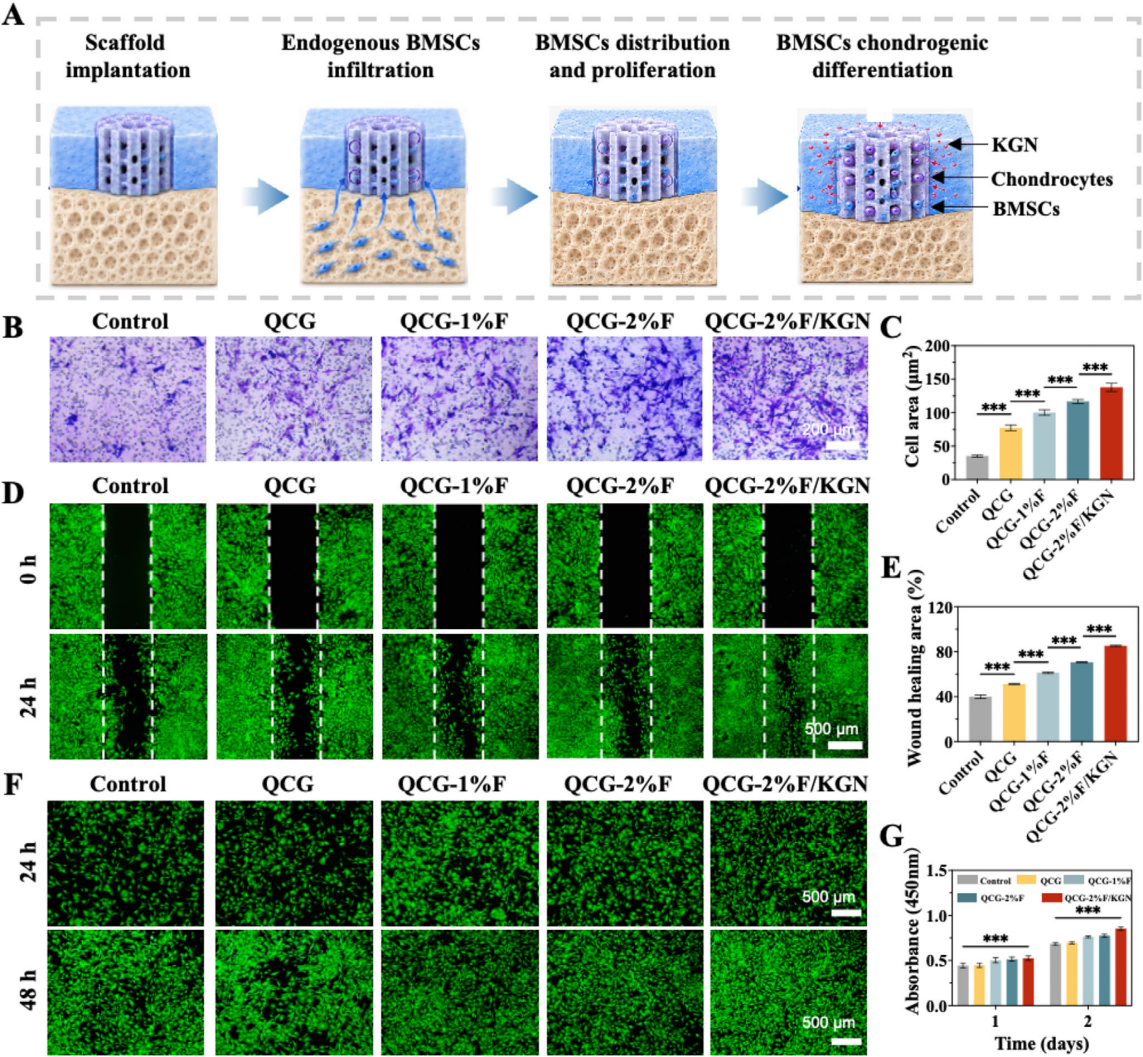
QCG‐2%F/KGN scaffolds maintain BMSC viability and enhance migratory activity in vitro. (A) Schematic illustrating the proposed sequential process after implantation: oriented channels provide a structurally permissive route for endogenous cell infiltration, the nanofiber‐reinforced microenvironment supports their distribution and proliferation, and sustained KGN release subsequently promotes chondrogenic differentiation. The schematic does not represent direct evidence of in vivo BMSC lineage or directional homing. (B,C) Representative crystal‐violet‐stained images of migrated BMSCs in the Transwell assay (B) and semi‐quantitative analysis of migrated cells (C) after treatment with Control medium or extracts from QCG, QCG‐1%F, QCG‐2%F, and QCG‐2%F/KGN scaffolds. QCG‐1%F and QCG‐2%F are drug‐free nanofiber‐containing groups. (D,E) Representative scratch‐wound images at 0 and 24 h (D) and quantitative analysis of wound closure (E), showing enhanced BMSC motility in the scaffold‐treated groups. White dashed lines indicate the wound boundaries. (F) Representative live/dead fluorescence images of BMSCs after 24 and 48 h of treatment with the indicated scaffold; viable cells are shown in green. (G) CCK‐8 analysis of BMSC metabolic activity/proliferation after 1 and 2 days of treatment. Scale bars: 200 µm in (B) and 500 µm in (D,F). Quantitative data are presented as mean ± SD (n = 5 independent biological experiments). One‐way ANOVA followed by Tukey's multiple‐comparison test was used for multi‐group comparisons; ^***^ *p* < 0.001; ns, not significant (*p* > 0.05).

Figure 4A summarizes the proposed biological sequence expected after implantation. The oriented channels provide a continuous structural route that may facilitate cell entry and distribution, the nanofiber‐reinforced walls support attachment and expansion, and sustained KGN release subsequently provides a chondrogenic cue. The Ttranswell assay demonstrated a formulation‐dependent increase in BMSC migration across the porous membrane (Figure 4B,C). Only a limited number of crystal‐violet‐positive cells crossed the membrane in the Control group, whereas QCG increased the number of migrated cells and the response became progressively stronger after incorporation of 1% and 2% nanofibers. Importantly, QCG‐1%F and QCG‐2%F in this assay contain 1 and 2 wt% drug‐free nanofibers, respectively; neither group contains KGN. Migration increased further from QCG‐1%F to QCG‐2%F, and QCG‐2%F/KGN produced the greatest response, increasing the count from approximately 35 cells per field in the Control group to approximately 137 cells per field. Thus, the assay supports enhanced BMSC migratory activity under the established in vitro conditions and distinguishes the contribution of drug‐free nanofiber reinforcement from the additional effect of KGN. The higher response of QCG‐2%F than QCG‐1%F is also consistent with the greater surface complexity and structural stability generated by the higher fiber content.

A scratch‐wound assay independently confirmed enhanced two‐dimensional motility (Figure 4D,E). At 0 h, the cell‐free gaps were comparable among groups. After 24 h, the Control group retained a broad open region, whereas the gap narrowed progressively in the QCG, QCG‐1%F, QCG‐2%F, and QCG‐2%F/KGN groups. Quantification showed that wound closure increased from 39.89 ± 1.57% in the Control group to 85.25 ± 0.58% in QCG‐2%F/KGN. The agreement between the Transwell and scratch assays is important because the two assays measure different aspects of migration: Transwell analysis requires cells to traverse a porous barrier under the imposed upper/lower chamber conditions, whereas scratch closure reflects coordinated lateral movement across a culture surface. The consistent group ranking therefore supports an increase in general migratory activity rather than an assay‐specific response, but it does not establish the in vivo origin or trajectory of the migrating cells.

Live/dead staining was performed at 24 and 48 h to determine whether the enhanced migration was accompanied by cytotoxicity (Figure 4F). Predominantly green fluorescence and very few dead cells were observed in all groups at both time points, with overall viability remaining close to 100%. Cell density increased from 24 to 48 h, particularly in the nanofiber‐containing groups. These observations indicate that neither residual processing components nor released KGN at the tested concentration caused overt acute toxicity. They also exclude the possibility that differences in migration arose simply from selective loss of cells in the less effective groups.

Cell Counting Kit‐8 (CCK‐8) analysis provided a quantitative measure of metabolic activity and proliferation (Figure 4G). Optical density increased from day 1 to day 2 in every group, confirming continued cell growth during exposure to the scaffold. At both time points, the scaffold‐treated groups showed values comparable to or higher than the Control group, and QCG‐2%F/KGN produced the highest response. The combination of near‐complete viability and increased CCK‐8 signal suggests that the composite scaffold did not merely preserve survival but created an in vitro environment that supported BMSC metabolic activity and expansion under the in vitro exposure conditions.

The migration‐promoting behavior can be interpreted in light of the material data. QCG and gelatin provide a hydrated, biocompatible matrix, while the nanofiber‐containing formulations exhibit improved structural stability and a greater capacity to retain fluid and soluble molecules. KGN has also been associated with mesenchymal stem cell motility and chondrogenic commitment. Collectively, the Transwell and scratch‐wound assays indicate that scaffold treatment enhances BMSC migratory activity in vitro. In parallel, the directionally aligned microchannels provide a continuous and relatively low‐resistance structural pathway that may facilitate endogenous cell infiltration, distribution, and retention within a defect. However, it is worth noting that these experiments do not directly establish the bone marrow origin, in vivo migration trajectory, or gradient‐dependent recruitment of the cells.

### 2.4 Nanofiber Reinforcement and Sustained KGN Release Enhance BMSC Colonization and Chondrogenic Differentiation

**FIGURE 5:**
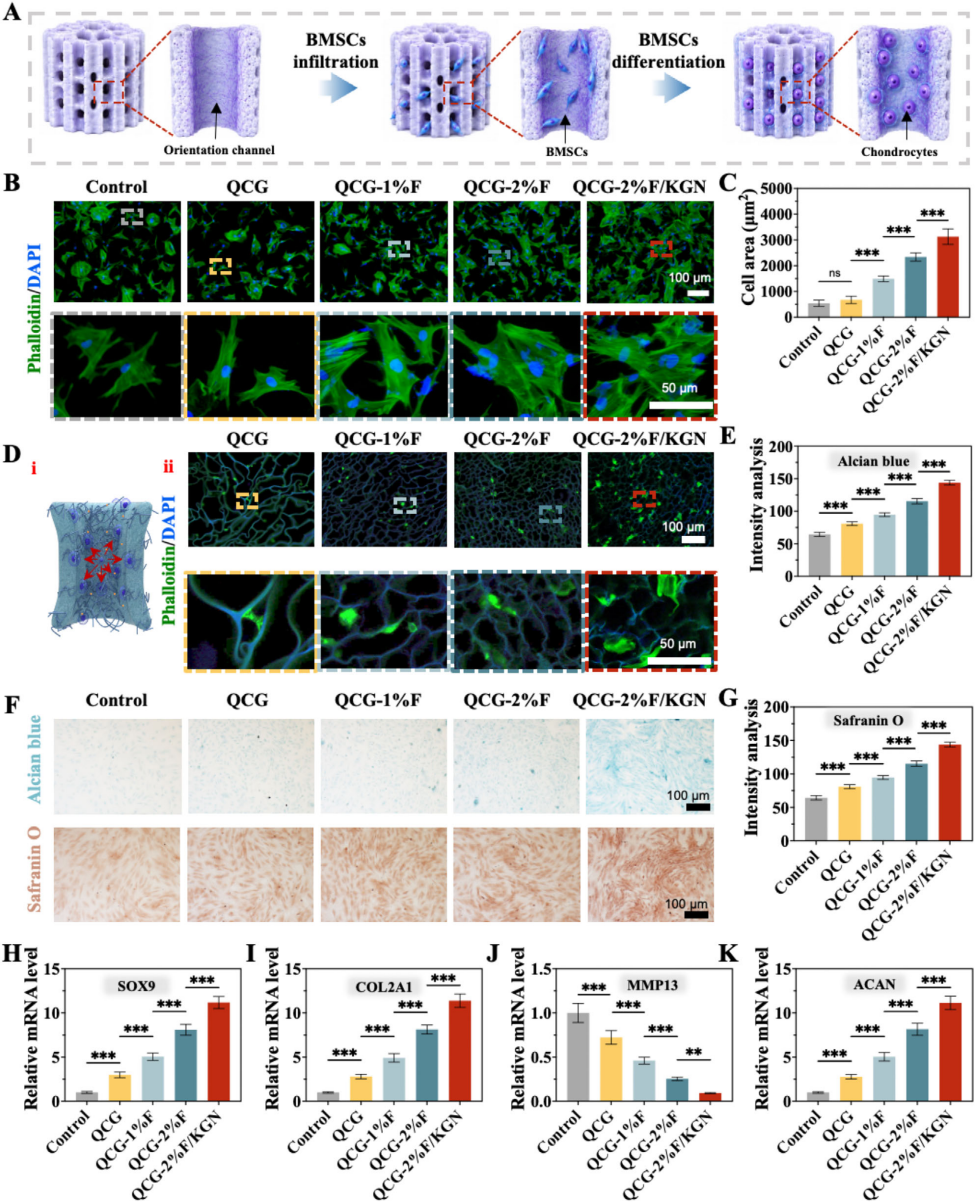
Nanofiber reinforcement and sustained KGN release enhance BMSC adhesion, spreading, and chondrogenic differentiation. (A) Schematic illustrating the proposed endogenous cell infiltration through the oriented microchannels and subsequent KGN‐mediated differentiation into chondrocytes within the scaffold. (B,C) Representative phalloidin/DAPI fluorescence images of BMSCs after indirect co‐culture with Control, QCG, QCG‐1%F, QCG‐2%F, and QCG‐2%F/KGN scaffolds (B), together with quantitative analysis of cell‐spreading area (C). The lower row in (B) shows enlarged views of the boxed regions. (D) Schematic showing BMSC colonization within the interconnected scaffold network (i), and representative confocal fluorescence images of phalloidin‐stained BMSCs directly cultured on QCG, QCG‐1%F, QCG‐2%F, and QCG‐2%F/KGN scaffolds (ii). The lower row shows enlarged views of the boxed regions. (E) Semi‐quantitative analysis of Alcian blue staining intensity. (F) Representative Alcian blue and Safranin O staining images of BMSCs after 14 days of chondrogenic induction with the indicated scaffold extracts. Alcian blue reflects glycosaminoglycan deposition, whereas Safranin O reflects cartilage proteoglycan accumulation. (G) Semi‐quantitative analysis of Safranin O staining intensity. (H–K) RT‐qPCR analysis of the chondrogenic and matrix‐remodeling genes (H) SOX9, (I) COL2A1, (J) MMP13, and (K) ACAN. QCG‐2%F/KGN treatment increased SOX9, COL2A1, and ACAN expression while suppressing MMP13 expression. Scale bars: 100 µm in the upper row and 50 µm in the enlarged row of (B); 100 µm in the upper row and 50 µm in the enlarged row of (D); and 100 µm in (F). Quantitative data are presented as mean ± SD (n = 5 independent biological experiments). One‐way ANOVA followed by Tukey's multiple‐comparison test was used for multi‐group comparisons; ^**^ *p* < 0.01, ^***^ *p* < 0.001; ns, not significant (*p* > 0.05).

The cell‐infiltration and differentiation concept is further illustrated in Figure 5A. After entering or colonizing the scaffold, BMSCs must spread over the pore walls, establish sustained cell‐material contacts, and deposit a cartilage‐specific ECM. Phalloidin/4′,6‐diamidino‐2‐phenylindole (DAPI) staining after indirect co‐culture revealed distinct differences in cytoskeletal organization (Figure 5B). Cells in the Control group remained relatively small and less extensively spread. QCG produced only a modest change, whereas QCG‐1%F and QCG‐2%F increased cell extension, actin‐filament organization, and the number of visible pseudopodia. The most pronounced spreading was observed in QCG‐2%F/KGN, where cells displayed broad, polygonal or spindle‐like morphologies and dense actin networks. Quantification confirmed that the mean spreading area increased from 541.80 ± 121.53 µm^2^ in the Control group to 3130.01 ± 301.23 µm^2^ in QCG‐2%F/KGN (Figure 5C). Direct culture on the scaffolds verified that these effects were maintained within the three‐dimensional architecture (Figure 5D). BMSCs adhered to all QCG‐based scaffolds, but the distribution and morphology differed substantially. On QCG, cells were relatively sparse and occupied limited portions of the smoother walls. Fiber‐containing scaffolds supported a denser, more interconnected cellular distribution, with cells extending along wall contours and across local junctions. QCG‐2%F/KGN showed the greatest apparent cell density, the longest pseudopodial extensions, and the broadest contact with the scaffold network. These findings correspond closely to the confocal and AFM data in Figure 2: exposed fibers and increased wall roughness provide more attachment points, while fiber bridges offer additional paths for cells to span neighboring structural units. The limited difference between Control and QCG compared with the marked increases after fiber incorporation indicates that the nanofiber‐derived micro/nanotopography, rather than the bulk polymer composition alone, was a major determinant of cell spreading.

Chondrogenic matrix formation was examined after 14 days. Semi‐quantitative analysis of Alcian blue staining showed a stepwise increase among the scaffold formulations (Figure 5E), and the representative images confirmed progressively stronger blue staining from Control to QCG‐2%F/KGN (Figure 5F). Alcian blue intensity increased from 53.78 ± 2.70 in the Control group to 119.82 ± 6.01 in QCG‐2%F/KGN, indicating a marked increase in sulfated glycosaminoglycan deposition. Safranin O staining showed the same overall pattern (Figure 5F,G): staining intensity rose from 64.37 ± 3.15 to 143.88 ± 3.62, with the strongest red‐orange matrix in the QCG‐2%F/KGN group. Because Alcian blue and Safranin O detect overlapping but not identical components of the cartilage matrix, their concordant increases provide stronger evidence of chondrogenic ECM accumulation than either assay alone.

Reverse transcription quantitative polymerase chain reaction (RT‐qPCR) further defined the differentiation state (Figure 5H–K). SOX9, a central regulator of chondrogenic commitment, increased progressively across the scaffold groups and reached its highest expression in QCG‐2%F/KGN (Figure 5H). COL2A1, the principal fibrillar collagen of hyaline cartilage, showed a similar graded increase (Figure 5I). In contrast, MMP13, a collagenase associated with cartilage matrix degradation and hypertrophic/catabolic remodeling, decreased as scaffold functionality increased and was lowest in QCG‐2%F/KGN (Figure 5J). ACAN, the major cartilage proteoglycan responsible for fixed‐charge density and water retention, was markedly upregulated in the KGN‐loaded group (Figure 5K). The combined expression pattern is more informative than any single marker. Increased SOX9 indicates activation of the chondrogenic transcriptional program; parallel increases in COL2A1 and ACAN demonstrate translation of this program into cartilage‐specific fibrillar and proteoglycan matrix; and suppression of MMP13 suggests that the newly deposited matrix was less exposed to catabolic degradation. This molecular profile is consistent with the stronger Alcian blue and Safranin O staining and argues against the possibility that enhanced staining resulted only from a larger number of nonspecifically proliferating cells.

Comparison of QCG‐2%F with QCG‐2%F/KGN clarifies the complementary roles of structure and biochemical signaling. The nanofiber‐reinforced scaffold substantially improved adhesion, spreading, and cell number, thereby increasing the population available for repair. However, the additional increases in SOX9, COL2A1, ACAN, and cartilage matrix staining after KGN loading show that cell accumulation alone was insufficient to produce the optimal phenotype. Sustained KGN release supplied a lineage‐specific cue over the same period in which the cells colonized the scaffold. Thus, the optimized scaffold operates sequentially: aligned channels provide structural access, nanofibers stabilize attachment and expansion, and KGN promotes chondrogenic differentiation of the colonizing BMSCs into matrix‐producing chondrogenic cells while limiting catabolic remodeling.

### 2.5 Shape‐adaptive QCG‐2%F/KGN Improves Macroscopic Defect Filling and Histological Cartilage Regeneration In Vivo

**FIGURE 6:**
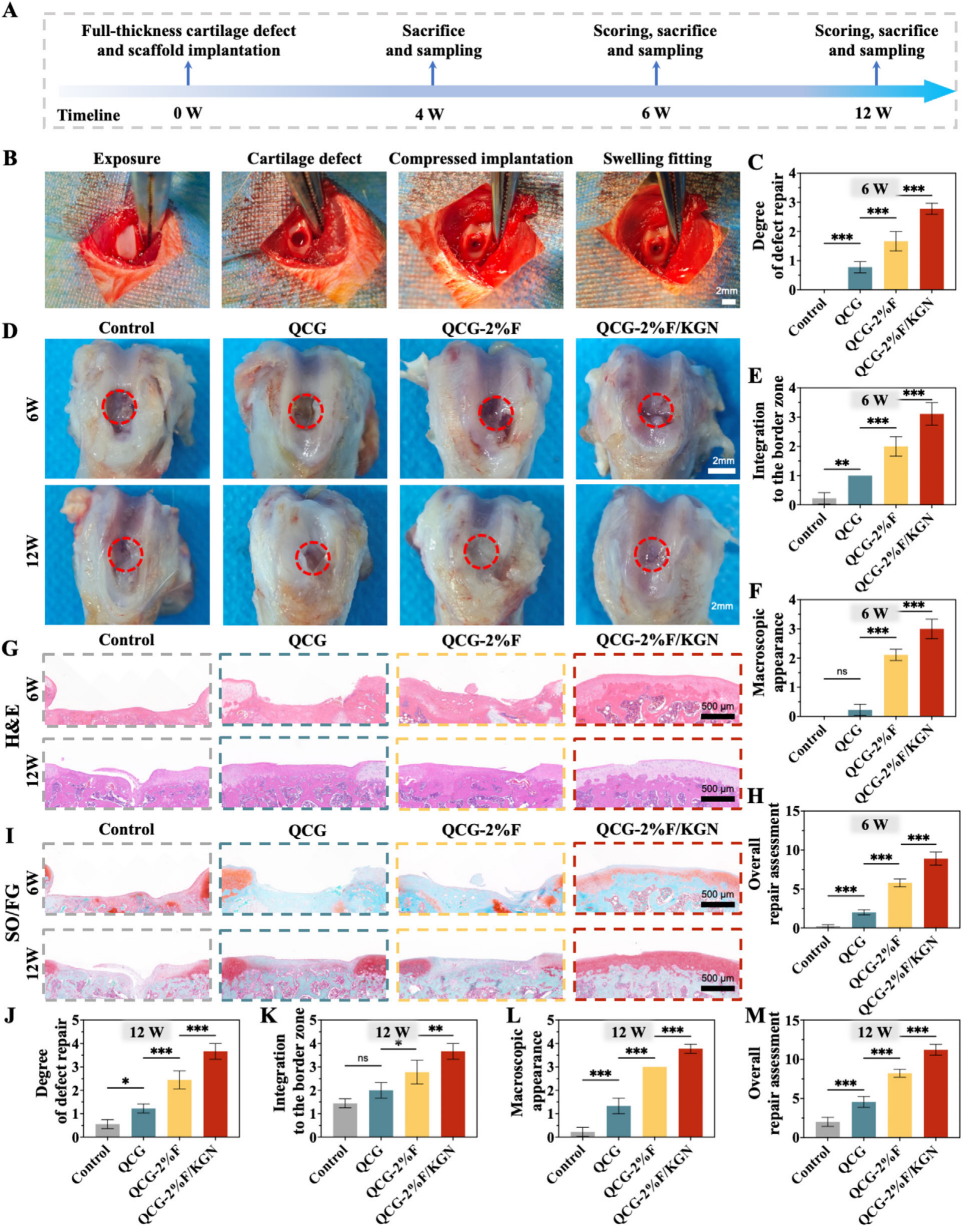
QCG‐2%F/KGN scaffolds improve macroscopic defect filling and histological cartilage regeneration in vivo. (A) Experimental timeline showing creation of the full‐thickness cartilage defect and scaffold implantation at week 0, tissue collection for transcriptomic analysis at week 4, and macroscopic scoring and tissue harvesting at weeks 6 and 12. (B) Representative surgical procedure, including exposure of the femoral trochlear groove, creation of the cartilage defect, implantation of the compressed scaffold, and liquid‐induced swelling and fitting within the defect. (C) ICRS score for the degree of defect repair at 6 weeks. (D) Representative gross images of cartilage defects in the Control, QCG, QCG‐2%F, and QCG‐2%F/KGN groups at 6 and 12 weeks. Red dashed circles indicate the original defect regions. (E) ICRS score for integration with the border zone at 6 weeks. (F) ICRS score for macroscopic appearance at 6 weeks. (G) Representative H&E‐stained sections of the defect regions at 6 and 12 weeks. (H) Overall ICRS macroscopic repair score at 6 weeks. (I) Representative Safranin O‐fast green‐stained sections at 6 and 12 weeks, showing proteoglycan‐rich cartilage matrix in red and subchondral bone in green. (J–M) ICRS scores at 12 weeks for the degree of defect repair (J), integration with the border zone (K), macroscopic appearance (L), and overall repair assessment (M). Scale bars: 2 mm in (B,D) and 500 µm in (G,I). Quantitative data are presented as mean ± SD (n = 5 animals per group; the individual animal was the experimental unit). One‐way ANOVA followed by Tukey's multiple‐comparison test was used for multi‐group quantitative comparisons; ^*^ *p* < 0.05, ^**^ *p* < 0.01, ^***^ *p* < 0.001; ns, not significant (*p* > 0.05).

The in vivo regenerative performance was evaluated using a full‐thickness trochlear cartilage‐defect model in rats (Figure 6A). Scaffolds were implanted at week 0, tissue from the defect region was collected at week 4 for transcriptomic analysis, and macroscopic and histological outcomes were assessed at weeks 6 and 12. This design allowed early molecular changes to be related to intermediate and later tissue‐level repair. The Control, QCG, QCG‐2%F, and QCG‐2%F/KGN groups also permitted separation of spontaneous repair, the effect of the directionally porous matrix, the contribution of nanofiber reinforcement, and the additional effect of sustained KGN release.

The surgical sequence in Figure 6B demonstrates the practical role of shape‐memory. After exposure of the femoral trochlear groove, a cylindrical full‐thickness defect was created and the scaffold was inserted in a compressed state. Contact with blood and tissue fluid then induced rapid swelling and recovery, allowing the scaffold to fill the defect and contact the surrounding cartilage without an auxiliary adhesive. This conformal fitting is important because interfacial gaps can permit scaffold displacement, interrupt cell migration from the defect base, and lead to discontinuous repair tissue. The implantation images therefore provide direct visual evidence that the hydration response characterized in vitro was retained during surgery.

At 6 weeks, the degree‐of‐defect‐repair score showed a clear stepwise improvement from Control to QCG, QCG‐2%F, and QCG‐2%F/KGN (Figure 6C). Gross images supported the scoring results (Figure 6D). The Control defects remained sharply demarcated and largely empty, with a deep central depression. QCG reduced the tendency toward defect enlargement and produced a flatter base, but the boundary remained obvious, and tissue filling was limited. QCG‐2%F supported appreciable neotissue formation, although a central depression and incomplete surface restoration were still evident. In contrast, QCG‐2%F/KGN produced substantially more complete filling, a shallower surface depression, and better continuity with the adjacent cartilage. These differences indicate that the directionally porous matrix alone can support some endogenous repair, whereas reinforcement and KGN are required for faster and more complete restoration.

The remaining 6‐week International Cartilage Repair Society (ICRS) subscores showed the same hierarchy. Integration with the border zone was poorest in Control, improved in QCG, increased further in QCG‐2%F, and was highest in QCG‐2%F/KGN (Figure 6E). Macroscopic appearance followed a similar trend (Figure 6F), with QCG‐2%F/KGN showing the smoothest and most cartilage‐like surface. The overall ICRS score integrated these features and confirmed a significant advantage of the optimized scaffold (Figure 6H). Improvement in integration is particularly important because a repair tissue that fills the central defect but remains separated from native cartilage is mechanically vulnerable and unlikely to provide durable function. The enhanced border integration may result from shape‐conformal fixation together with better cell retention and matrix production at the scaffold‐cartilage interface.

Hematoxylin and eosin (H&E) staining revealed marked differences in tissue organization (Figure 6G). At 6 weeks, the Control defect was occupied mainly by sparse, disorganized fibrous tissue and retained a pronounced surface discontinuity. QCG produced more tissue, but the repair remained thin and irregular. QCG‐2%F increased the amount of neotissue and improved continuity, consistent with enhanced structural support and cell colonization, yet the tissue still lacked the uniform architecture of native cartilage. QCG‐2%F/KGN already showed a more continuous cartilage‐like layer with better integration at the defect margins. By 12 weeks, all groups exhibited some progression, but Control and QCG still contained irregular fibrous repair and incomplete restoration, QCG‐2%F displayed a thicker but partly heterogeneous layer, and QCG‐2%F/KGN showed the thickest and most continuous cartilage‐like tissue with a comparatively smooth articular surface. Safranin O‐fast green staining assessed proteoglycan‐rich cartilage matrix and subchondral bone organization (Figure 6I). At 6 weeks, the Control, QCG, and QCG‐2%F groups showed extensive unstained or weakly stained regions, indicating limited cartilage proteoglycan deposition. QCG‐2%F/KGN exhibited a larger red‐stained area within the defect, demonstrating earlier formation of cartilage‐like matrix. At 12 weeks, staining increased in the scaffold groups, but the quality remained distinct. QCG and QCG‐2%F contained substantial repair tissue with heterogeneous or relatively weak Safranin O staining, consistent with a fibrocartilage‐rich phenotype. QCG‐2%F/KGN displayed the strongest and most spatially continuous staining, with better continuity between regenerated and native cartilage. This finding indicates that the optimized scaffold improved not only defect filling but also the biochemical composition and maturation of the repair tissue.

Gross evaluation at 12 weeks further confirmed sustained improvement (Figure 6D). The Control and QCG groups retained visible defects, irregular surfaces, and incomplete connection to surrounding cartilage. QCG‐2%F achieved better filling and border continuity, but a residual surface difference remained. The QCG‐2%F/KGN defect was largely occupied by smooth, glossy tissue, and the original boundary was difficult to distinguish. Quantitative scores for degree of repair, border integration, macroscopic appearance, and overall repair were all highest in QCG‐2%F/KGN (Figure 6J–M). The persistence of group differences at 12 weeks indicates that the early advantage was not merely caused by transient swelling of the scaffold; rather, it developed into structurally and biochemically superior tissue regeneration.

The comparison among groups separates the effects of the individual design elements. QCG provided aligned channels and hydration‐driven defect filling, which improved repair relative to untreated defects but did not generate mature cartilage. Addition of 2% short fibers stabilized the scaffold, increased adhesive interfaces, and promoted tissue ingrowth, explaining the intermediate performance of QCG‐2%F. Nevertheless, this group still exhibited residual depressions, heterogeneous staining, and fibrocartilage‐like repair. Only QCG‐2%F/KGN combined efficient colonization with a sustained differentiation signal, resulting in better filling, integration, surface morphology, and proteoglycan deposition. The in vivo data therefore support a sequential mechanism in which shape‐memory recovery secures the scaffold, the reinforced architecture provides an environment favorable for endogenous cell infiltration and retention, and KGN improves the phenotype and maturation of the newly formed tissue. Although the in vivo findings consistently favored QCG‐2%F/KGN across the evaluated repair outcomes, the study was conducted with five animals per group and no formal a priori power calculation was performed. The present cohort therefore supports the major treatment effects observed here but may be less sensitive to smaller between‐group differences or inter‐animal variability. Larger, prospectively powered studies will be important to further confirm the reproducibility of these findings and strengthen their translational relevance.

The in vivo design did not include a QCG + free/bolus KGN comparator. Therefore, the present data establish that the core–shell formulation provides sustained local KGN release and improves repair relative to the tested matrix and nanofiber controls, but they do not directly demonstrate superiority over simple local administration of free KGN. Prior coaxial‐electrospinning studies have shown that core–shell architectures can regulate diffusion and prolong release of encapsulated bioactive agents, including KGN, which supports the delivery rationale used here [38, 39, 40]. A direct free‐KGN comparison will be required to quantify the incremental benefit of core–shell‐mediated release in this specific scaffold.

### 2.6 QCG‐2%F/KGN Promotes Hyaline Cartilage Matrix Formation While Suppressing Fibrotic and Catabolic Remodeling

**FIGURE 7:**
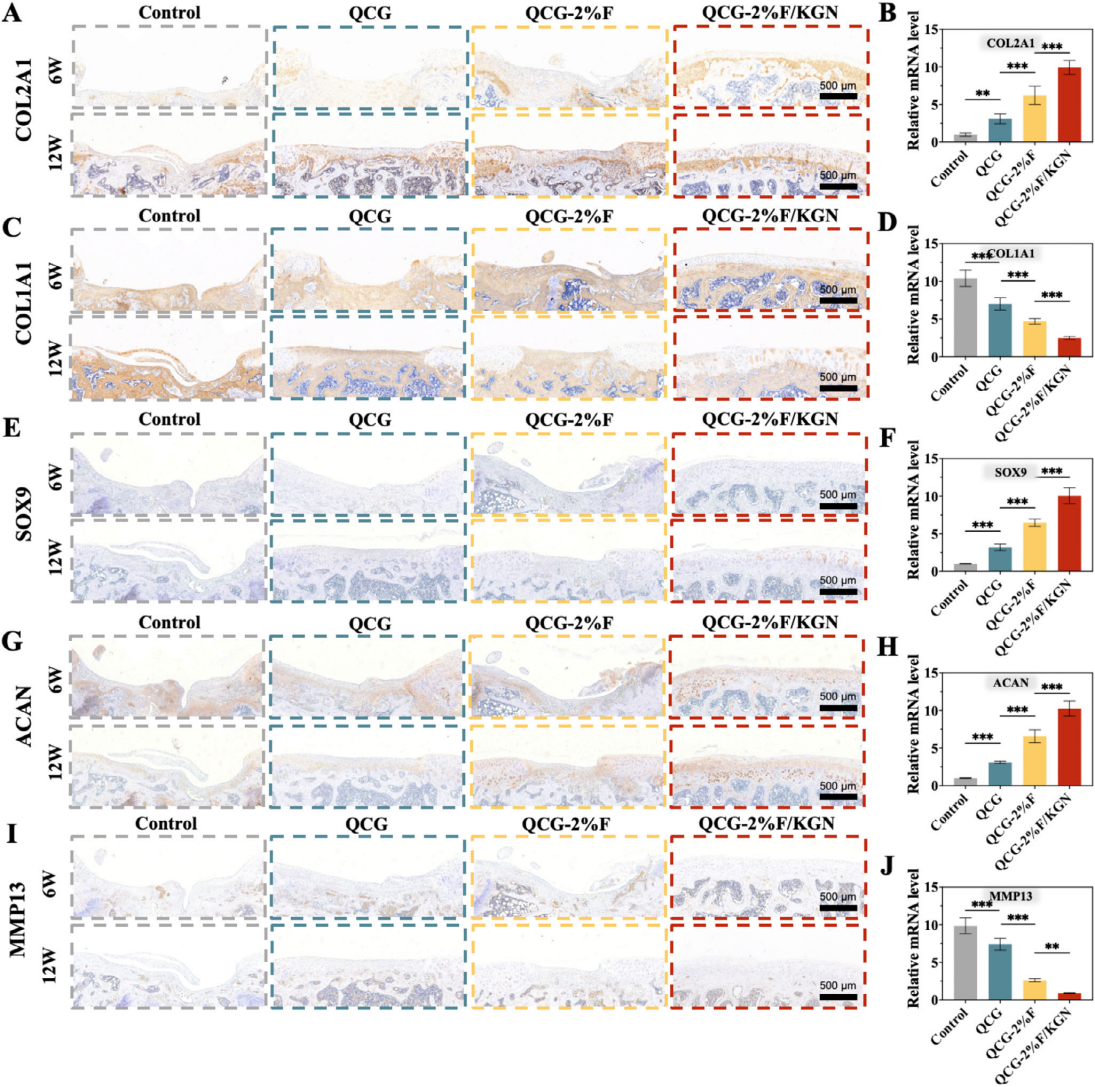
QCG‐2%F/KGN promotes hyaline cartilage matrix formation while suppressing fibrotic and catabolic remodeling in the defect region. (A,B) Representative type II collagen (COL2A1) immunohistochemical staining at 6 and 12 weeks (A) and corresponding RT‐qPCR analysis of COL2A1 expression at 12 weeks (B). (C,D) Representative type I collagen (COL1A1) immunohistochemical staining (C) and corresponding RT‐qPCR analysis of COL1A1 expression at 12 weeks (D). (E,F) Representative SOX9 immunohistochemical staining (E) and corresponding RT‐qPCR analysis of SOX9 expression at 12 weeks (F). (G,H) Representative ACAN immunohistochemical staining (G) and corresponding RT‐qPCR analysis of ACAN expression (H). (I,J) Representative MMP13 immunohistochemical staining (I) and corresponding RT‐qPCR analysis of MMP13 expression at 12 weeks (J). Brown DAB staining indicates positive protein expression, and nuclei are counterstained blue. Compared with the other groups, QCG‐2%F/KGN treatment increased COL2A1, SOX9, and ACAN expression and reduced COL1A1 and MMP13 expression, supporting enhanced hyaline cartilage formation and reduced fibrocartilage formation and matrix degradation. Scale bars: 500 µm in (A,C,E,G,I). Quantitative data are presented as mean ± SD (n = 5 animals per group; the individual animal was the experimental unit). One‐way ANOVA followed by Tukey's multiple‐comparison test was used for multi‐group RT‐qPCR comparisons; ^**^ *p* < 0.01, ^***^ *p* < 0.001; ns, not significant (*p* > 0.05).

To determine whether the regenerated tissue represented hyaline cartilage or fibrous/fibrocartilaginous repair, immunohistochemistry and RT‐qPCR were performed for cartilage‐anabolic, fibrotic, and catabolic markers (Figure 7). COL2A1 was first examined as a defining component of hyaline cartilage. COL2A1 staining was weak and spatially limited in the Control and QCG groups at 6 weeks, increased in QCG‐2%F, and was strongest and most continuous in QCG‐2%F/KGN (Figure 7A). At 12 weeks, COL2A1 deposition increased in the scaffold‐treated defects, but the optimized group retained the largest positive area and the most cartilage‐like distribution. RT‐qPCR showed the same group ordering, with significantly higher COL2A1 expression after QCG‐2%F/KGN treatment (Figure 7B). The agreement between staining and transcript levels indicates that the stronger signal reflected active cartilage‐matrix synthesis rather than passive retention of pre‐existing collagen.

Type I collagen provided an opposing indicator of fibrous or fibrocartilaginous repair. Collagen type I alpha 1 chain (COL1A1) staining was extensive in the Control group and remained prominent in QCG and QCG‐2%F, particularly within irregular repair regions (Figure 7C). QCG‐2%F/KGN showed substantially less COL1A1‐positive tissue at both 6 and 12 weeks. Correspondingly, COL1A1 mRNA decreased progressively across the treatment groups and was lowest in the optimized scaffold group (Figure 7D). The reciprocal relationship between high COL2A1 and low COL1A1 is more convincing than either marker alone because it demonstrates a shift in matrix identity from scar‐like collagen toward hyaline cartilage collagen.

SOX9 staining and gene expression were then used to evaluate chondrogenic lineage regulation. Few SOX9‐positive cells were detected in the Control defect, whereas scaffold implantation increased the signal and QCG‐2%F/KGN produced the strongest response (Figure 7E,F). The increase was evident at 6 weeks and remained detectable at 12 weeks. SOX9 is required for activation and maintenance of key cartilage genes, including COL2A1 and ACAN. Its sustained expression therefore provides a mechanistic link between KGN‐mediated differentiation and the enhanced cartilage‐specific matrix observed histologically. The weaker SOX9 response in QCG‐2%F also explains why improved cell colonization in that group did not fully translate into hyaline cartilage formation.

Aggrecan deposition showed a complementary matrix‐anabolic pattern. ACAN immunostaining was sparse in the Control and QCG groups, increased after nanofiber reinforcement, and became more extensive within the QCG‐2%F/KGN repair tissue (Figure 7G). RT‐qPCR confirmed significant ACAN upregulation in the scaffold‐treated groups, with a marked increase after KGN‐containing treatment (Figure 7H). Because ACAN binds water and generates the osmotic swelling pressure required for cartilage compressive function, its increase is directly relevant to tissue quality rather than merely serving as a generic differentiation marker. The ACAN pattern is also consistent with the intense Safranin O staining in Figure 6I.

MMP13 was examined to assess matrix‐catabolic remodeling. Stronger MMP13‐positive staining was present in the Control and QCG defects, while the signal decreased in QCG‐2%F and was lowest in QCG‐2%F/KGN (Figure 7I). Gene‐expression analysis confirmed significant suppression of MMP13 in the optimized group (Figure 7J). This reduction is important because newly deposited COL2A1‐rich matrix will not accumulate effectively if collagen degradation remains high. The scaffold therefore appears to improve cartilage repair through a dual action: increasing anabolic matrix production while simultaneously reducing a major matrix‐degrading enzyme.

The time‐dependent staining patterns further clarify tissue maturation. At 6 weeks, QCG‐2%F/KGN already combined strong SOX9 staining with early COL2A1/ACAN deposition with low COL1A1 and MMP13, indicating that cells within the repair tissue had entered a chondrogenic rather than fibrotic program. By 12 weeks, the greater continuity and intensity of COL2A1 and ACAN were accompanied by continued suppression of COL1A1 and MMP13, suggesting maturation and preservation of the newly formed cartilage matrix. In contrast, the other groups showed some tissue filling but retained a matrix profile dominated to a greater extent by type I collagen and catabolic remodeling.

Together, Figure 7 confirms that the superior macroscopic and histological outcome of QCG‐2%F/KGN was not simply due to a larger volume of repair tissue. QCG provided a porous structural route for cell access within the defect, and nanofibers increased cell anchoring and tissue occupancy; however, without KGN, the resulting tissue remained comparatively fibrocartilaginous. Sustained KGN release shifted the balance toward SOX9‐driven COL2A1 and ACAN synthesis while suppressing COL1A1 and MMP13. The optimized scaffold therefore promoted both the formation and preservation of a hyaline cartilage‐like ECM.

### 2.7 Transcriptomic Analysis Reveals Coordinated Regulation of Chondrogenesis, Matrix Anabolism, and Inflammation

RNA sequencing was performed on tissue collected from the defect regions of the Control and QCG‐2%F/KGN groups at 4 weeks to investigate molecular events preceding the clear histological differences observed at 6 and 12 weeks. The 4‐week time point is particularly informative because endogenous cells have entered the defect and begun differentiation, while the repair tissue has not yet fully matured. The transcriptomic data therefore provide a snapshot of the early regenerative microenvironment established by the scaffold rather than simply describing the final tissue phenotype.

**FIGURE 8:**
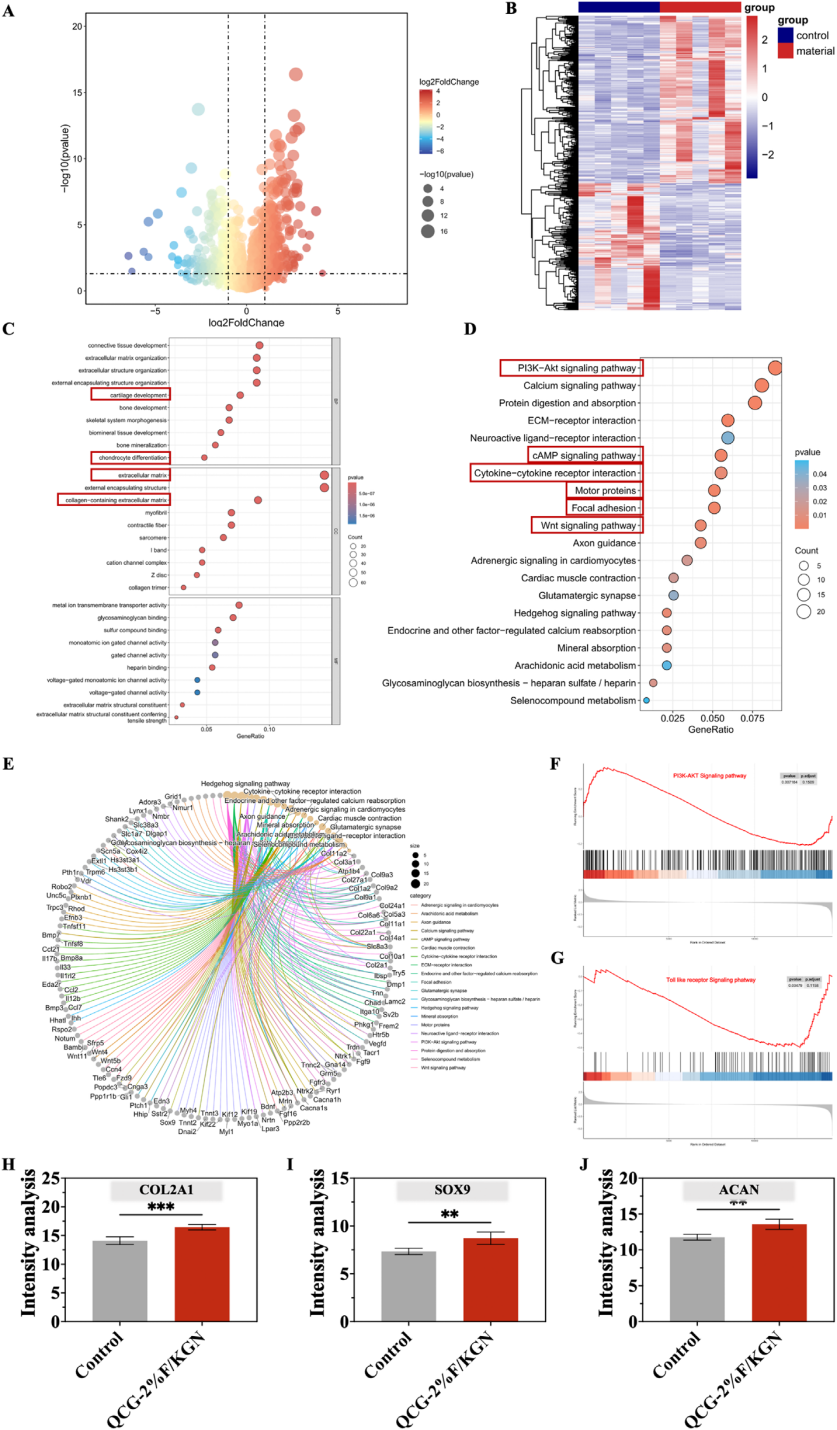
Transcriptomic analysis shows coordinated enrichment of chondrogenic and matrix‐anabolic programs and suppression of inflammatory signaling after QCG‐2%F/KGN treatment. Cartilage tissues were collected from the defect regions of the Control and QCG‐2%F/KGN groups at 4 weeks after implantation for RNA sequencing (n = 5 per group). (A) Volcano plot of differentially expressed genes (DEGs) between the two groups; QCG‐2%F/KGN treatment resulted in 309 upregulated and 234 downregulated genes relative to the Control group. (B) Hierarchical‐clustering heatmap showing the expression patterns of the identified DEGs across individual samples. (C) Gene Ontology enrichment analysis showing significant enrichment of biological processes and cellular components associated with cartilage development, chondrocyte differentiation, extracellular matrix (ECM) organization, and collagen‐containing ECM. (D) KEGG pathway enrichment analysis highlighting repair‐related pathways, including PI3K‐AKT, cAMP, cytokine‐cytokine receptor interaction, focal adhesion, Wnt, and other cell‐survival and matrix‐regulatory pathways. Bubble size represents the number of enriched genes, and bubble color represents the enrichment p value. (E) Chord diagram showing associations between representative DEGs and enriched KEGG pathways. (F) Gene set enrichment analysis (GSEA) showing positive enrichment of the PI3K‐AKT signaling pathway in the QCG‐2%F/KGN group. (G) GSEA showing negative enrichment of Toll‐like receptor‐associated signaling, consistent with attenuation of inflammatory and matrix‐catabolic responses. (H–J) Normalized expression levels of the cartilage‐associated genes (H) COL2A1, (I) SOX9, and (J) ACAN, all of which were significantly increased after QCG‐2%F/KGN treatment. Data in (H–J) are presented as mean ± SD (n = 5 animals per group; individual animal as the experimental unit). Two‐group comparisons in (H–J) used an unpaired two‐tailed Student's t‐test; ^**^ *p* < 0.01, ^***^ *p* < 0.001.

The volcano plot identified a broad treatment‐associated transcriptional response (Figure 8A). Relative to Control, QCG‐2%F/KGN produced 309 significantly upregulated and 234 significantly downregulated differentially expressed genes (DEGs). The presence of substantial changes in both directions indicates active reprogramming rather than a nonspecific global increase in transcription. Hierarchical clustering of these DEGs clearly separated the Control and material‐treated samples (Figure 8B), while samples within each group displayed similar expression patterns. This separation supports the reproducibility of the scaffold‐induced response and shows that the early molecular environment had already diverged before mature cartilage formation was evident.

Gene Ontology (GO) enrichment analysis linked the DEGs to biological processes and cellular components directly relevant to cartilage repair (Figure 8C). Prominent terms included cartilage development, chondrocyte differentiation, ECM organization, extracellular structure organization, and collagen‐containing ECM. Enrichment of these terms is consistent with the later increases in SOX9, COL2A1, and ACAN and with the proteoglycan‐rich tissue observed histologically. It should be emphasized that overrepresentation analysis identifies biological themes represented among the DEGs; it does not by itself establish that every gene within an enriched term changed in the same direction. Nevertheless, the convergence of multiple cartilage‐ and matrix‐related categories strongly indicates that QCG‐2%F/KGN redirected the defect environment toward a regenerative program.

Kyoto Encyclopedia of Genes and Genomes (KEGG) analysis showed that the DEGs were distributed across several interacting pathways rather than a single dominant mechanism (Figure 8D). Enriched pathways included phosphoinositide 3‐kinase/protein kinase B (PI3K‐AKT) signaling, cyclic adenosine monophosphate (cAMP) signaling, focal adhesion, Wnt signaling, cytokine‐cytokine receptor interaction, motor proteins, ECM‐receptor interaction, and calcium‐associated signaling. These pathways correspond to distinct stages of the proposed repair sequence. Focal adhesion, ECM‐receptor interaction, and motor proteins are consistent with cellular responses to aligned and roughened scaffold surfaces; PI3K‐AKT and cAMP signaling are associated with survival, proliferation, and matrix synthesis; Wnt signaling influences lineage progression and cartilage maturation; and cytokine‐related pathways reflect remodeling of intercellular communication within the injured joint.

The chord diagram further illustrated that individual DEGs were connected to multiple enriched pathways and that each pathway shared genes with other functional modules (Figure 8E). This network‐like organization is important because the scaffold simultaneously changes physical contact, drug exposure, cell composition, and inflammatory signaling. The repair response would therefore not be expected to depend on a single linear pathway. Instead, the chord relationships support coordinated regulation of cell‐matrix adhesion, motility, differentiation, matrix production, and immune communication.

Gene set enrichment analysis (GSEA) provided directional information for two key pathway‐associated gene sets. PI3K‐AKT signaling was positively enriched in QCG‐2%F/KGN tissue (Figure 8F), an association that is compatible with the greater cell number and anabolic matrix production observed in vitro and in vivo. In contrast, Toll‐like receptor (TLR) signaling was negatively enriched (Figure 8G), which is consistent with a less inflammatory and less catabolic defect environment. These enrichment patterns do not establish that either pathway is causally required for the regenerative effect; rather, they identify pathway‐level associations that align with the observed increase in COL2A1 and ACAN and the reduction in MMP13.

Expression analysis of representative cartilage genes independently supported the pathway‐level findings. COL2A1 was significantly increased in the QCG‐2%F/KGN group (Figure 8H), SOX9 was upregulated (Figure 8I), and ACAN also showed a significant increase (Figure 8J). These early transcriptional changes preceded the strong protein deposition detected at later time points, providing a temporal connection between the 4‐week molecular response and the 6‐ and 12‐week histological outcomes. Their agreement with the RT‐qPCR and immunohistochemical data also strengthens confidence that the sequencing results reflect genuine cartilage‐specific regeneration.

Western blotting and densitometric quantification were performed to further validate signaling pathways highlighted by the transcriptomic analysis (Figure S8). The QCG‐2%F/KGN group showed increased p‐AKT/AKT and p‐CREB/CREB ratios, together with changes in cyclin D1, β‐catenin, and PKA expression, compared with the Control group. These protein‐level changes were broadly consistent with the transcriptomic enrichment of PI3K‐AKT, cAMP/CREB, and Wnt‐related signaling pathways. However, because the present analysis was performed using repair tissues containing multiple cell populations and without pathway‐specific pharmacological or genetic perturbation, these findings should be interpreted as correlative evidence rather than definitive proof of pathway activation, inhibition, or causal dependence.

Integrating the omics data with the preceding material and biological results suggests a multistage model rather than a proven linear mechanism. Liquid‐induced shape recovery establishes contact with the defect, while the aligned channels and nanofiber‐reinforced surfaces provide a structural environment favorable for cell entry, adhesion, and expansion. Sustained KGN exposure is associated with increased SOX9, COL2A1, and ACAN and reduced MMP13. PI3K‐AKT‐ and cAMP/CREB‐related changes are consistent with a survival/anabolic phenotype, whereas negative enrichment of TLR‐associated signaling is consistent with reduced inflammatory/catabolic activity. Tissue from the defect contains multiple cell populations, so the identified genes cannot be assigned exclusively to BMSCs or newly differentiated chondrocytes. Another point that remains to be clarified is the cellular origin of the reparative cells. The Transwell and scratch assays demonstrate enhanced BMSC migratory activity in vitro, and the aligned microchannels provide a structurally permissive route for cell infiltration; however, these observations do not directly establish that marrow‐resident BMSCs migrate through the scaffold toward the cartilage defect in vivo. Future lineage‐tracing or in vivo cell‐tracking studies will therefore be needed to define the source and migration trajectory of the cells contributing to repair. The concordance between transcriptomic enrichment and western blot/densitometric findings supports pathway‐level association, but does not establish definitive pathway dependence.

## 3 Conclusions

In this study, we developed a liquid‐responsive shape‐memory QCG‐2%F/KGN scaffold by integrating DFC with KGN‐loaded core–shell nanofiber reinforcement. The aligned porous architecture provides a structurally permissive route for cell infiltration and distribution, the interpenetrating nanofiber network stabilizes the pore walls and creates ECM‐like adhesion interfaces, and hydration‐triggered shape recovery enables compressed implantation followed by conformal defect filling. Meanwhile, sustained KGN release was associated with enhanced BMSC chondrogenic differentiation in vitro and with increased SOX9, COL2A1, and ACAN together with reduced COL1A1 and MMP13 in the repair tissue. These coordinated functions improved macroscopic integration, proteoglycan deposition, and hyaline cartilage‐like regeneration in vivo. Transcriptomic and representative protein data further identified PI3K‐AKT‐, cAMP/CREB‐, Wnt‐, and TLR‐related changes that are consistent with the observed regenerative phenotype, but do not establish pathway causality. Overall, the scaffold enhanced BMSC proliferation, migratory activity, and chondrogenic differentiation in vitro, while its directionally aligned channels may favor endogenous cell infiltration and colonization in vivo. The bone marrow origin and migration trajectory of these cells remain to be established.

## 4 Materials and Methods

### 4.1 Materials

Polycaprolactone (PCL; average Mn = 80 000) and poly(vinyl alcohol) (PVA; Mw ≈ 87 000 Da; 99% hydrolyzed) were purchased from Sigma‐Aldrich. Silk fibroin (SF) was purchased from Cellamatrix Co., Ltd. (Wuhan, China). Quaternized chitosan (QCS; degree of quaternization, 98%; Mw ≈ 10 kDa), gelatin, and KGN were purchased from Macklin (China). All chemicals were of analytical grade and used as received.

### 4.2 Preparation of Core–Shell Nanofibers

SF and PCL were dissolved in hexafluoroisopropanol (HFIP) at a mass ratio of 4:1 to prepare a 10 wt% shell solution. For the core solution, KGN was added to a 10 wt% PVA solution at 10 mg/mL and stirred at 80°C for 24 h. Coaxial electrospinning was performed at 15 kV with a 15 cm needle‐to‐collector distance. The shell and core flow rates were 1.0 and 0.3 mL/h, respectively. The collected nanofiber mats were vacuum‐dried for 72 h to remove residual solvent.

### 4.3 Fabrication of Scaffolds

Nanofiber mats were fragmented by high‐speed homogenization and incorporated into a 2 wt% quaternized chitosan/gelatin solution (QCG; QCS/gelatin mass ratio, 1:1) at nanofiber contents of 0, 1, or 2 wt%. An additional 3 wt% fiber formulation was prepared for mechanical screening only and was not used in the biological experiments. Each mixture was homogenized for 10 min, followed by the addition of genipin and a further 10 min of homogenization. The mixtures were crosslinked at 37°C for 24 h, directionally frozen using liquid nitrogen, and vacuum‐dried.

The resulting scaffolds were designated QCG, QCG‐1%F, QCG‐1%F/KGN, QCG‐2%F, and QCG‐2%F/KGN. QCG denotes the nanofiber‐free scaffold. QCG‐1%F and QCG‐2%F contain 1 and 2 wt% drug‐free nanofibers, respectively, whereas QCG‐1%F/KGN and QCG‐2%F/KGN contain KGN‐loaded nanofibers at the corresponding contents. Thus, references to QCG‐1%F and QCG‐2%F in the migration and cell‐response experiments specifically denote drug‐free nanofiber groups.

### 4.4 Structural and Morphological Characterization

The morphology of the nanofibers and scaffolds was examined by field‐emission SEM (Sigma, Zeiss, Germany). Nanofiber diameter and scaffold channel size were measured using ImageJ [41, 42]. The core–shell structure was examined by TEM (JEM‐2100, JEOL, Japan). Nanofiber distribution within the scaffolds was visualized by confocal laser scanning microscopy, and pore‐wall topography was evaluated by AFM (SPM‐9700).

### 4.5 Porosity Measurement

Scaffolds were cut into regular cubes. The scaffold volume was measured as *V*, and the dry mass was recorded as *M1*. The samples were immersed in ethanol for 24 h to allow complete ethanol infiltration, and the mass was rapidly measured and recorded as *M2*. The pore volume (*V*_0_) and porosity were calculated as follows [43]:

(1) $$V_{0}=\frac{M_{2}-M_{1}}{\rho_{\mathrm{ethanol}}}$$

(2) $$\mathrm{Porosity}\;(\%)\;=\frac{V_{0}}{V}\;\times 100\%$$

### 4.6 Swelling Ratio Test

The initial scaffold volume was measured as *V*_1_. After soaking in phosphate‐buffered saline (PBS) for 48 h to reach equilibrium, the volume was measured as *V*_2_. The swelling ratio was calculated as:

(3) $$\mathrm{Swelling}\;\mathrm{ratio}\;(\%)\;=\frac{V_{2}}{V_{1}}\;\times 100\%$$

### 4.7 Water Absorption Measurement

The maximum water absorption was first determined. The initial mass of the scaffold was recorded as *W*_1_. After soaking in PBS for 48 h, the mass was measured as *W*_2_. For cyclic water absorption, the water‐saturated scaffold was compressed to 50% of its volume and then re‐immersed in PBS for water absorption measurement. This procedure was repeated for ten cycles. Water absorption was calculated as:

(4) $$\mathrm{Water}\;\mathrm{absorption}\;(\%)\;=\frac{W_{2}-W_{1}}{W_{1}}\;\times 100\%$$

### 4.8 Water Retention Measurement

Water retention was determined as follows. The initial dry mass of the scaffold was recorded as *W*. After soaking in PBS for 48 h, the mass was recorded as *W*_1_. The samples were then centrifuged at 500 r/min for 5 min and weighed to obtain *W*_2_. For cyclic water retention, the centrifuged scaffold was re‐soaked in PBS and then centrifuged again at 500 r/min, and the mass was recorded; the procedure was repeated for ten cycles. Water retention was calculated as:

(5) $$\mathrm{Water}\;\mathrm{retention}\;(\%)\;=\frac{W_{2}-W}{W_{1}-W}\;\times 100\%$$

### 4.9 In Vitro KGN Release

Scaffolds were placed in centrifuge tubes containing PBS and incubated on a temperature‐controlled shaker (37°C, 100 rpm). At predetermined time points, 1 mL of PBS was collected and replaced with 1 mL of fresh PBS. Drug‐free scaffolds with the same composition were incubated in PBS under identical conditions and used as blank controls. At each sampling time point, the absorbance of the corresponding blank medium was subtracted from that of the KGN‐containing release sample to minimize background absorption arising from polymer leachates or degradation products. The blank‐corrected absorbance was measured at 282 nm using a UV‐vis spectrophotometer [27]. KGN standard solutions of known concentration were measured under the same detection conditions to establish the calibration curve. KGN concentration and cumulative release were then calculated from the blank‐corrected signal. No independent HPLC validation was performed in the present study.

### 4.10 In Vitro Degradation

Dry scaffolds were weighed (W1) and incubated in phosphate‐buffered saline (PBS) at 37°C. At each time point, the samples were removed, rinsed with deionized water, dried at 60°C for 48 h, and weighed again (W2). The residual‐mass percentage was calculated using the following equation:

(6) $$\mathrm{Residual}\;\mathrm{mass}\left(\%\right)=\frac{w_{2}}{w_{1}}\;*100\%$$

### 4.11 Mechanical Properties

Mechanical properties were measured using a universal testing machine. Scaffolds were cut into square specimens and tested under dry and hydrated conditions in both the longitudinal direction (parallel to the aligned channels) and the transverse direction (perpendicular to the channels). For additional mechanical screening, hydrated QCG and QCG‐2%F/KGN scaffolds were subjected to 500 repeated compression cycles to assess fatigue‐related structural stability. A 3 wt% fiber formulation was also evaluated in wet‐state compression to determine whether further fiber addition produced a stable deformation response.

### 4.12 Live/Dead Staining and CCK‐8 Assay

BMSCs (Servicebio Co., Ltd., Wuhan, China) were cultured in complete growth medium at 37°C in a humidified atmosphere with 5% CO_2_ and 95% humidity. The medium was changed every 2 days, and passage 3–4 BMSCs were used for all in vitro experiments.

Scaffolds were sterilized by ethanol immersion and rinsed three times with PBS. The scaffolds were then incubated in complete medium for 24 h to obtain extracts of different scaffolds. Cytotoxicity was evaluated using a live/dead staining kit (Solarbio Science & Technology Co., Ltd., Beijing, China). BMSCs (2 × 10^5^ cells per well) were seeded in 24‐well plates and cultured for 12 h to allow attachment, followed by incubation with the corresponding scaffold extracts for 24 h. The cells were then stained with the live/dead reagents and observed under a fluorescence microscope.

A Cell Counting Kit‐8 assay (CCK‐8; Solarbio Science & Technology Co., Ltd., Beijing, China) was further performed to assess the effect of scaffold extracts on BMSC viability. BMSCs (2 × 10^3^ cells per well) were seeded in 96‐well plates and cultured for 24 and 48 h, followed by treatment with different scaffold extracts; complete medium served as the control. The optical density (OD) at 450 nm was measured at the end of each exposure period using a microplate reader (Multiskan Spectrum, Thermo Scientific, USA) to evaluate cell number.

### 4.13 Transwell Migration Assay

A Transwell assay was performed to evaluate the effect of scaffolds on cell migration. BMSCs were serum‐starved for 24 h prior to seeding. Scaffolds were placed in the lower chamber, and 2 × 10^4^ BMSCs were seeded into the upper chamber. After 24 h, the Transwell insert was removed, washed three times with PBS, fixed with 4% paraformaldehyde (PFA) for 30 min, washed again with PBS three times, and stained with 0.1% crystal violet for 30 min. Non‐migrated cells were gently removed using a cotton swab. Migrated cells were quantified using ImageJ.

### 4.14 Scratch Wound Assay

A scratch assay was conducted to assess the effect of scaffolds on cell motility. BMSCs (2 × 10^5^ cells per well) were seeded in 6‐well plates and cultured until ∼90% confluence. A uniform scratch was created using a 200 µL pipette tip, and the wells were washed with PBS to remove detached cells. Different scaffold extracts were then added. After 24 h of culture, cells were stained using the live/dead kit and observed under an inverted microscope. The wound closure area was quantified using ImageJ.

### 4.15 Cytoskeletal Staining

Cytoskeletal staining was used to evaluate the effect of scaffolds on cell growth. A 24‐well plate equipped with cell culture inserts was used for indirect co‐culture. BMSCs were seeded onto the bottom of each well at a density of 2 × 10^5^ cells per well, while scaffolds were cut into small disks and placed in the inserts. After 24 h, the cytoskeleton was stained with phalloidin, and the nuclei were stained with 4′,6‐diamidino‐2‐phenylindole (DAPI). Stained cells were observed under an inverted microscope, and cell spreading area was quantified using ImageJ.

### 4.16 Cell Morphology and Adhesion

Scaffolds were cut into regular squares and placed at the bottom of 24‐well plates. BMSCs were seeded onto each scaffold at a density of 2 × 10^5^ cells per scaffold. After 24 h, scaffolds were thoroughly rinsed with PBS and fixed with 4% paraformaldehyde for 30 min. The cytoskeleton was stained with phalloidin, and the scaffolds and cells were observed by confocal fluorescence microscopy.

### 4.17 Chondrogenic Differentiation

A 6‐well transwell plate was used. BMSCs were seeded in the lower chamber at a density of 2 × 10^5^ cells per well, and the scaffolds were placed in the upper chamber. The medium was changed every 2 days. After 14 days, scaffolds were removed, and the lower chamber was rinsed three times with PBS, fixed with 4% paraformaldehyde for 30 min, and then stained with Alcian blue and Safranin O.

### 4.18 RNA and qRT‐PCR

Total RNA was extracted from cells and tissues using TRIzol reagent (Vazyme, R401‐01, China). For each sample, 1000 ng of RNA was reverse‐transcribed using HiScript II Q RT SuperMix for qPCR (+gDNA wiper; Vazyme, R223‐01, China). Quantitative reverse‐transcription polymerase chain reaction (RT‐qPCR) was performed using Taq Pro Universal SYBR qPCR Premix (Vazyme, Q712‐02, China) and a LightCycler 480 system (Roche, USA). Relative gene expression was normalized to 18S ribosomal RNA or glyceraldehyde‐3‐phosphate dehydrogenase (GAPDH) and calculated using the 2−ΔΔCt method. Primer sequences are listed in Table S1.

### 4.19 In Vivo Implantation

Male Sprague‐Dawley (SD) rats weighing 250–300 g were used for orthotopic implantation (n = 5 per group). Animals were randomly assigned to the Control, QCG, QCG‐2%F, or QCG‐2%F/KGN group. All procedures followed the National Research Council Guide for the Care and Use of Laboratory Animals and were approved by the Institutional Animal Care and Use Committee of the Animal Experimental Center of Wuhan University (No. WP20250040). Anesthesia and perioperative care were used to minimize pain and distress.

After anesthesia, the femoral trochlear groove was exposed through a medial knee incision. A full‐thickness defect measuring 2 mm in diameter and 1.5 mm in depth was created in the proximal trochlear groove using a biopsy punch. The corresponding scaffold was compressed, implanted into the defect, and allowed to hydrate and recover its shape. The wound was closed in layers, and the animals were monitored postoperatively.

Rats were euthanized at 6 and 12 weeks after surgery. Femoral condyles were harvested and evaluated using the International Cartilage Repair Society (ICRS) macroscopic scoring system. Samples were fixed in 4% paraformaldehyde for 48 h, decalcified for 4 weeks, dehydrated, embedded in paraffin, and sectioned at 4 µm. Sections were subjected to hematoxylin and eosin (H&E) staining, Safranin O‐fast green staining, and immunohistochemical staining for COL2A1, COL1A1, SOX9, ACAN, and MMP13 (all from Abcam, Cambridge, UK; 1:400).

### 4.20 Western Blot Analysis

To validate the signaling pathways identified by transcriptomic analysis, cartilage‐defect tissues were collected from the different groups at 4 weeks after implantation. The samples were immediately frozen in liquid nitrogen and stored at −80°C until analysis. Total proteins were extracted by homogenizing the tissues in radioimmunoprecipitation assay (RIPA) lysis buffer supplemented with protease and phosphatase inhibitors. The lysates were incubated on ice for 30 min and centrifuged at 12 000 × g for 15 min at 4°C. The resulting supernatants were collected, and protein concentrations were determined using a bicinchoninic acid assay.

Equal amounts of protein were separated by sodium dodecyl sulfate‐polyacrylamide gel electrophoresis and transferred onto polyvinylidene fluoride membranes. After blocking with 5% non‐fat milk or bovine serum albumin in Tris‐buffered saline containing 0.1% Tween‐20 for 1 h at room temperature, the membranes were incubated overnight at 4°C with primary antibodies against phosphorylated AKT (p‐AKT), total AKT, cyclin D1, β‐catenin, phosphorylated CREB (p‐CREB), total CREB, protein kinase A (PKA), and GAPDH. Bovine serum albumin was used for blocking membranes incubated with phosphoprotein antibodies. After three washes with TBST, the membranes were incubated with the corresponding horseradish peroxidase‐conjugated secondary antibodies for 1 h at room temperature. Protein bands were visualized using an enhanced chemiluminescence detection system. The expression of p‐AKT and p‐CREB was normalized to total AKT and total CREB, respectively, whereas cyclin D1, β‐catenin, and PKA expression was normalized to GAPDH. For target‐protein detection, the transferred PVDF membranes were physically sectioned according to the expected molecular‐weight ranges before primary‐antibody incubation and imaging.

### 4.21 Transcriptomic Sequencing

Tissue from the cartilage‐defect region was collected 4 weeks after implantation (n = 5 per group), snap‐frozen in liquid nitrogen, and stored at −80°C. Total RNA was extracted and quality‐controlled. After transcriptome‐library construction, sequencing was performed on an Illumina NovaSeq 6000 platform (Personalbio, Nanjing, China). Differentially expressed genes were subjected to GO, KEGG, and GSEA.

### 4.22 Statistical Analysis

Quantitative data are presented as mean ± standard deviation (SD). Data were reviewed for technical integrity before analysis, and biological replicates were not excluded post hoc solely on the basis of statistical extremity. Molecular data were normalized only as specified in the corresponding methods; no additional transformation was applied unless stated. Statistical analyses were performed using GraphPad Prism 11.0.0. For material characterization, n denotes independently prepared scaffold samples; for in vitro assays, n denotes independent biological experiments; and for in vivo analyses, n denotes animals, with the individual animal treated as the experimental unit. Technical replicates and multiple readouts obtained from the same biological specimen were not treated as additional independent biological replicates. Two‐group comparisons used an unpaired two‐tailed Student's t‐test. Multiple‐group comparisons used one‐way analysis of variance (ANOVA) followed by Tukey's multiple‐comparison post hoc test. The statistical test and n are specified in each quantitative figure legend. The significance level was α = 0.05. Significance indicators are defined as ^*^ *p* < 0.05, ^**^ *p* < 0.01, and ^***^ *p* < 0.001; ns indicates no statistically significant difference (*p* > 0.05).

## Author Contributions

Jiyang Zeng performed the majority of the experiments, conducted data analysis, and drafted the initial version of the manuscript. Yawei Li, Zhiming Tu, Hong Ma, Yuliang Dai, and Zhaoling Ma participated in scaffold fabrication and characterization, in vitro and in vivo experiments, histological evaluation, transcriptomic analysis, and scientific discussion. Wei Li, Tao Yuan, and Bing Wang conceived and designed the study, interpreted the experimental results, revised the manuscript, and supervised the overall project. All authors reviewed and approved the final version of the manuscript.

## Conflicts of Interest

The authors declare no conflicts of interest.

## Supporting information




**Supporting File**: advs77845‐sup‐0001‐SuppMat.docx.

## Data Availability

Research data are not shared.
