# Supplementary material for: Liquid‐Responsive Shape‐Memory Nanofiber‐Reinforced Scaffolds for Cartilage Repair
Source: Adv Sci (Weinh). 2026 Sep 27:e77845. Online ahead of print. doi: 10.1002/advs.77845 (PMC13616390; doi:10.1002/advs.77845)
Supplement: Supplementary file 1 [file ADVS-9999-e77845-s001.docx]

**Additional information**

Supplementary Information is available for this paper.

**Supplementary Materials for**

**Liquid-Responsive Shape-Memory Nanofiber-Reinforced Scaffolds for Cartilage Repair**

Jiyang Zeng ^a, b^, Wei Li ^c, *^, Yawei Li ^a, b^, Zhiming Tu ^a, b, Hong Ma^ ^a, b^, Yuliang Dai ^a, b^, Zhaoling Ma ^d^, Tao Yuan ^a, b *^, Bing Wang ^a, b *^

^a^ Department of Spine Surgery, Second Xiangya Hospital of Central South University, Changsha 410011, China.

^b^ Hunan Digital Spine Research Institute, Department of Science and Technology of Hunan Province, Changsha 410011, China.

^c^ Hubei Key Laboratory of Biomass Resource Chemistry and Environmental Biotechnology, Hubei International Scientific and Technological Cooperation Base of Sustainable Resource and Energy, School of Resource and Environmental Science, Wuhan University, Wuhan 430079, China.

^d^ Guangxi Key Laboratory of Low Carbon Energy Materials, Guangxi Normal University, Guilin, 541004, China.

* Corresponding authors:

E-mail addresses: [yuantao_65@csu.edu.cn](mailto:yuantao_65@csu.edu.cn) (T. Yuan); [liwei418@whu.edu.cn](mailto:liwei418@whu.edu.cn) (W. Li); [wbxyeyy@csu.edu](mailto:wbxyeyy@csu.edu).cn (B. Wang)

**Figure S1. Quantification of the mean channel size of QCG, QCG-1%F/KGN, and QCG-2%F/KGN scaffolds.** Channel dimensions were measured from representative SEM images using ImageJ. Data are presented as mean ± SD (n = 5 independent scaffold samples). One-way ANOVA followed by Tukey's multiple-comparison test was used; ns, not significant (*p* > 0.05).

**Figure S2. Confocal fluorescence microscopy of the nanofiber-free QCG scaffold.** Representative three-dimensional reconstruction and corresponding enlarged image show the aligned porous architecture of the QCG scaffold in the absence of incorporated short nanofibers. Scale bars: 500 μm and 100 μm.

**Figure S3. Atomic force microscopy characterization of the nanofiber-free QCG scaffold.** Representative two-dimensional and three-dimensional AFM topographic images of the pore-wall surface show a relatively smooth surface with a height variation of approximately 1.19 μm over a 20 × 20 μm scanning area. Scale bar: 10 μm.

**Figure S4. In vitro degradation profile of the QCG-2%F/KGN scaffold.** Residual scaffold mass was monitored during incubation in PBS at 37 °C for 30 days. The scaffold exhibited gradual degradation without rapid structural collapse, retaining approximately 95% of its initial mass during the first 10 days and approximately 80% at day 30. Data are presented as mean ± SD (n = 5 independent scaffold samples); this single-group degradation time course is reported descriptively.

 **Figure S5. Dry-state anisotropic compressive properties of the nanofiber-reinforced scaffolds.** (A, B) Representative longitudinal compressive stress-strain curves of QCG, QCG-1%F/KGN, and QCG-2%F/KGN scaffolds under dry conditions (A) and corresponding maximum compressive stress (B). (C, D) Representative transverse compressive stress-strain curves under dry conditions (C) and corresponding maximum compressive stress (D). Increasing the short-nanofiber content markedly enhanced the dry-state load-bearing capacity of the scaffolds in both testing directions. Data are presented as mean ± SD (n = 5 independent scaffold samples). One-way ANOVA followed by Tukey's multiple-comparison test was used for group comparisons; ****p* < 0.001; ns, not significant (*p* > 0.05).

**Figure S6. Wet-state compressive stress–strain behavior of scaffolds with different nanofiber contents.** (A) Representative longitudinal compressive stress–strain curves of QCG-1%F/KGN, QCG-2%F/KGN, and QCG-3%F/KGN scaffolds. (B) Representative transverse compressive stress–strain curves of the corresponding formulations. Increasing the nanofiber content to 3% increased the peak compressive stress but did not result in a uniformly improved deformation response, with an apparent instability at high compressive strain. In comparison, the 2% formulation exhibited a smoother mechanical response over the same deformation range, supporting its selection for subsequent experiments.

**Figure S7. Wet-state cyclic compressive behavior of QCG and QCG-2%F/KGN scaffolds under repeated loading.** (A, B) Representative longitudinal cyclic-compression stress profiles of QCG (A) and QCG-2%F/KGN (B) scaffolds over 500 loading cycles. (C, D) Representative transverse cyclic-compression stress profiles of QCG (C) and QCG-2%F/KGN (D) scaffolds over 500 loading cycles. The nanofiber-free QCG scaffold showed progressive loss of mechanical resistance during repeated compression, whereas QCG-2%F/KGN maintained a comparatively stable cyclic response in both loading directions, supporting improved fatigue resistance after nanofiber reinforcement.

**Figure S8. Western blot analysis and densitometric quantification of repair-associated signaling pathways.** (A) Representative western blot images of phosphorylated AKT (p-AKT), total AKT, and GAPDH in the Control, QCG, QCG-2%F, and QCG-2%F/KGN groups. (B) Densitometric quantification of the p-AKT/AKT ratio. (C) Densitometric quantification of cyclin D1 expression normalized to GAPDH. (D) Representative western blot images of cyclin D1, β-catenin, and GAPDH associated with Wnt signaling. (E) Densitometric quantification of β-catenin expression normalized to GAPDH. (F) Densitometric quantification of the p-CREB/CREB ratio. (G) Representative western blot images of phosphorylated CREB (p-CREB), total CREB, PKA, and GAPDH associated with cAMP signaling. (H) Densitometric quantification of PKA expression normalized to GAPDH. Data are presented as mean ± SD (n = 5 independent biological samples). Statistical significance was determined by one-way ANOVA followed by Tukey’s multiple-comparison test. ns, not significant; *p < 0.05, **p < 0.01, ***p < 0.001.

**Table S1. Primer sequences used for RT-qPCR.**

| **Gene** | **Primer sequence (5′–3′)** | **Amplicon length (bp)** |
| --- | --- | --- |
| SOX9 | F: CGTCAACGGCCCCAGCA  R: TGCGCCCCACACCATGA | 69 |
| COL2A1 | F: CCTGAAACTCTGCCACCCAG  R: GTTCTTCCGAGGCAGTCG | 151 |
| COL1A1 | F: ACCCCAAGGAGAAGAAGCAT  R: AGGTTGCCAGTCTGTTGGTC | 202 |
| ACAN | F: ACACCCCTACCCTTGCTTCT  R: AAAGTGTCCAAGGCATCCAC | 124 |
| MMP13 | F: CCCTGGAGCCCTGATGTTT  R: CTCTGGTGTTTTGGGGTGCT | 109 |
| GAPDH | F: CTCAACTACATGGTCTACATGTTCCA  R: AAGAATGGGAGTTGCTGTTGAAGTC | 81 |
